# The Challenging Pathway of Treatment for Neurogenesis Impairment in Down Syndrome: Achievements and Perspectives

**DOI:** 10.3389/fncel.2022.903729

**Published:** 2022-05-11

**Authors:** Fiorenza Stagni, Renata Bartesaghi

**Affiliations:** ^1^Department for Life Quality Studies, University of Bologna, Rimini, Italy; ^2^Department of Biomedical and Neuromotor Sciences, University of Bologna, Bologna, Italy

**Keywords:** Down syndrome, neurogenesis, gliogenesis, dendritic development, pharmacotherapy, mouse models

## Abstract

Down syndrome (DS), also known as trisomy 21, is a genetic disorder caused by triplication of Chromosome 21. Gene triplication may compromise different body functions but invariably impairs intellectual abilities starting from infancy. Moreover, after the fourth decade of life people with DS are likely to develop Alzheimer’s disease. Neurogenesis impairment during fetal life stages and dendritic pathology emerging in early infancy are thought to be key determinants of alterations in brain functioning in DS. Although the progressive improvement in medical care has led to a notable increase in life expectancy for people with DS, there are currently no treatments for intellectual disability. Increasing evidence in mouse models of DS reveals that pharmacological interventions in the embryonic and neonatal periods may greatly benefit brain development and cognitive performance. The most striking results have been obtained with pharmacotherapies during embryonic life stages, indicating that it is possible to pharmacologically rescue the severe neurodevelopmental defects linked to the trisomic condition. These findings provide hope that similar benefits may be possible for people with DS. This review summarizes current knowledge regarding (i) the scope and timeline of neurogenesis (and dendritic) alterations in DS, in order to delineate suitable windows for treatment; (ii) the role of triplicated genes that are most likely to be the key determinants of these alterations, in order to highlight possible therapeutic targets; and (iii) prenatal and neonatal treatments that have proved to be effective in mouse models, in order to rationalize the choice of treatment for human application. Based on this body of evidence we will discuss prospects and challenges for fetal therapy in individuals with DS as a potential means of drastically counteracting the deleterious effects of gene triplication.

## Introduction

Down syndrome (DS) is a relatively high-incidence pathology (∼1 in every 800–1,000 live births; see Antonarakis et al., 2020; Hughes-McCormack et al., 2020) caused by triplication of Hsa21. Increased expression of Hsa21 genes (and genes on other chromosomes) impairs development and functions of various organs, including the brain (Bull, 2020). While some disorders may not be present in all individuals with DS, intellectual disability (ID) is the invariable hallmark of DS (Zigman, 2013; Ballard et al., 2016; Lott and Head, 2019). ID scores range from moderately (IQ of 50–70) to severely (IQ of 20–35; Bull, 2020) affected; even in its milder form, intellectual performance may compromise the ability to live independently. ID is already detectable in children with DS, especially regarding language, memory, and adaptive behavior, and is exacerbated with age (Godfrey and Lee, 2020). Moreover, individuals with DS are at a high risk of developing Alzheimer’s disease (AD) after 40 years of age (Zigman and Lott, 2007). There is currently no treatment for ID in DS.

A reduction in the number of neurons forming the brain and in brain size are typical phenotypic features of DS starting from prenatal life stages (see Stagni et al., 2018). Although there is no simple correlation between neocortical size and cognitive abilities, cortical expansion during primate evolution is thought to underlie the extraordinary cognitive abilities of humans (Kaas, 2019). Thus, it is very likely that the reduced number of neurons in the DS brain is a key determinant of the ID that characterizes this pathology.

The reduction in neuron number in DS is not due to neuronal degeneration but to impairment in the process of neurogenesis during fetal life, the critical period during which almost all neurons that form the brain are generated. Accumulating evidence clearly shows that neurogenesis reduction in DS is attributable to two main causes: cell cycle alterations, leading to a reduced proliferation potency of neural progenitor cells (NPCs), and augmented differentiation of the daughter cells into glial elements at the expense of their differentiation into neuronal cells (thus, reduction of neurogenesis, i.e., generation of new neurons, *sensu stricto*; see Stagni et al., 2018). Moreover, the process of neuron maturation (dendritogenesis) in early infancy is also impaired (Takashima et al., 1981, 1994), causing defective connectivity. This knowledge poses an intriguing question: is it possible and feasible to restore or to improve neurogenesis in DS with early and targeted interventions? A positive answer to this question would imply the possibility of preventing ID in individuals with DS, the more optimistic of hypotheses, or at least of boosting brain functioning, a more cautious hypothesis. This challenge requires knowledge of the mechanisms that underlie neurogenesis alterations in DS and preclinical evidence in DS mouse models that neurogenesis and cognition can be pharmacologically ameliorated. Studies carried out during the past 20 years in mouse models have shown that treatments during adult life stages aimed at ameliorating cognitive performance are promising, thus encouraging this effort (see Costa and Scott-McKean, 2013; Gardiner, 2015; Stagni et al., 2015a; Hart et al., 2017; Vacca et al., 2019; Rueda et al., 2020a). Fewer studies have examined the effects of treatment during the early neonatal and embryonic period (see (Stagni et al., 2015a and section “Achievements Obtained by Early Pharmacotherapies in Down Syndrome Models”). Thanks to these studies, we now know that it is possible to fully restore neurogenesis impairment with precocious interventions. Moreover, both prenatal and neonatal treatment lead to restoration not only of neurogenesis but also of dendritic maturation, connectivity, and cognitive performance. This body of evidence provides proof of principle demonstration that neurogenesis can be pharmacologically ameliorated in DS, and may spur the scientific community to continue in its search for pharmacological treatments that are effective and applicable to people with DS.

In this review, we will summarize achievements and challenges in the field of treatment for neurogenesis impairment in DS by focusing on the following issues: (i) Overview of the timeline of neurogenesis in humans as a tool to understand DS-related alterations; (ii) Spatiotemporal characteristics of neurogenesis alterations in DS. This knowledge provides fundamental information regarding the window/s of opportunity for treatment; (iii) Current knowledge of the genetic and cellular mechanisms responsible for neurogenesis impairment in DS, as revealed by human and mouse model studies. This knowledge is fundamental for the design of treatments; (iv) Achievements obtained so far through early pharmacotherapies in DS models; and (v) Long-term perspectives for treatment, with particular emphasis on the necessity to rationalize our efforts in the identification of the more suitable therapeutic targets and treatments.

## Overview of the Timeline of Neurogenesis, Gliogenesis, and Neuron Maturation in the Normal Brain

The brain is formed by neurons, astrocytes, and oligodendrocytes, plus microglia which is the nervous immune system. The generation of neurons, astrocytes, and oligodendrocytes takes place during definite and partially overlapping phases of brain development. Key steps of brain development are outlined below (mainly based on Rakic, 2009; Rice and Barone, 2010; Stiles and Jernigan, 2010; Yamaguchi et al., 2016; Kostović et al., 2019), as being instrumental in understanding pathological changes in DS. The prenatal period comprises the embryonic period, that goes from conception to gestational week (GW) 8 and the fetal period, that goes from GW9 to birth. Neurogenesis begins in the embryonic period and continues to mid-gestation. The neural stem cells (also called NPCs) appear during gastrulation, a process that takes place between embryonic day (E) 14 and E21. The first brain structure is the neural tube, a hollow cavity that begins to form at E20–E27 and will subsequently give origin to the different parts of the nervous system. The inner surface of the neural tube is lined with NPCs. This region is called the ventricular zone (VZ) because the cavity of the neural tube will give origin to the cerebral ventricles. The VZ is gradually replaced by the subventricular zone (SVZ).

### Neurogenesis

**FIGURE 1:**
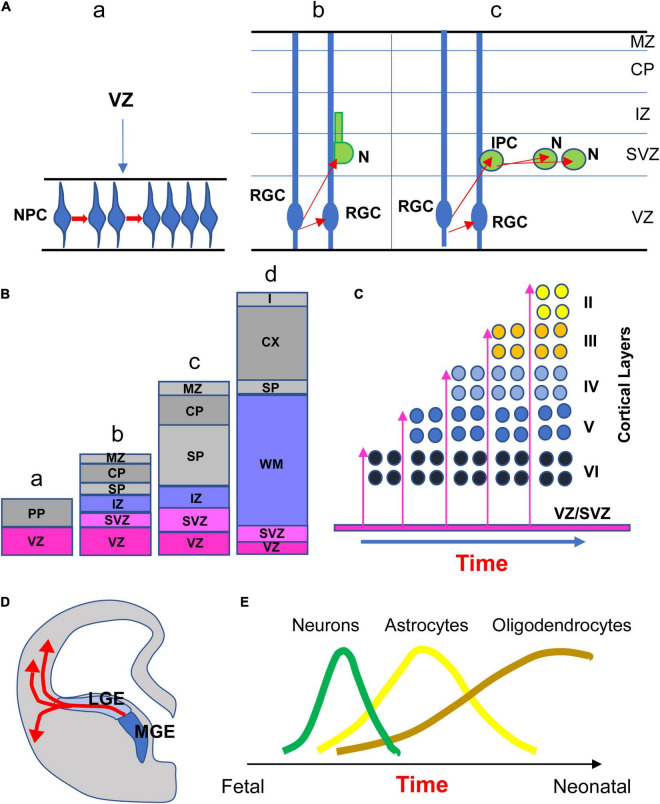
**(A)** The first step of neurogenesis increases the size of the pool of the neural progenitor cell (NPC) population through symmetrical cell divisions (a). NPCs, that begin as neuroepithelial cells, then become radial glia cells (RGC). Beginning at ∼E42, asymmetrical divisions of RGC produce one RGC that remains proliferative and one neuron (N). The postmitotic neuron leaves the proliferative zone migrating along radial glia processes to reach its place in the developing cortex (b). Asymmetric divisions of RGC can produce one RGC that remains proliferative and one intermediate progenitor cell (IPC; also called transit amplifying cell) that undergoes divisions (c). **(B)** Development of laminar compartments in the neocortical cerebral wall from early embryonic (a) to late fetal period (d). Neurons migrate radially from the VZ out to the developing cortex. The first neurons leaving the VZ form the preplate (PP; a). Further waves of neuron migration split the PP into the marginal zone (MZ) and the subplate (SP), giving origin to a new region called the cortical plate (CP; b, c) which will give origin to the future cortex (d). The CP is separated from the SVZ by the intermediate zone (IZ), that will later become the white matter layer (d). **(C)** The earliest neurons migrating to the cortical plate settle to what will become layer VI. Successively migrating neurons settle to progressively more superficial cortical layers (inside-out pattern). **(D)** Interneurons born in the ganglionic eminences reach their destination through tangential migration. **(E)** Relative timing of neurogenesis, astrogliogenesis, and oligogliogenesis during brain development in humans, schematically depicted based on evidence in rodents. Abbreviations: CP, cortical plate; IPC, intermediate progenitor cell; IZ, intermediate zone; LGE, lateral ganglionic eminence; MGE, medial ganglionic eminence; MZ, marginal zone; N, neuron; NPC, neural progenitor cells; PP, preplate; RGC, radial glial cells; SP, subplate; SVZ, subventricular zone; VZ, ventricular zone; and WM, white matter.

**FIGURE 2:**
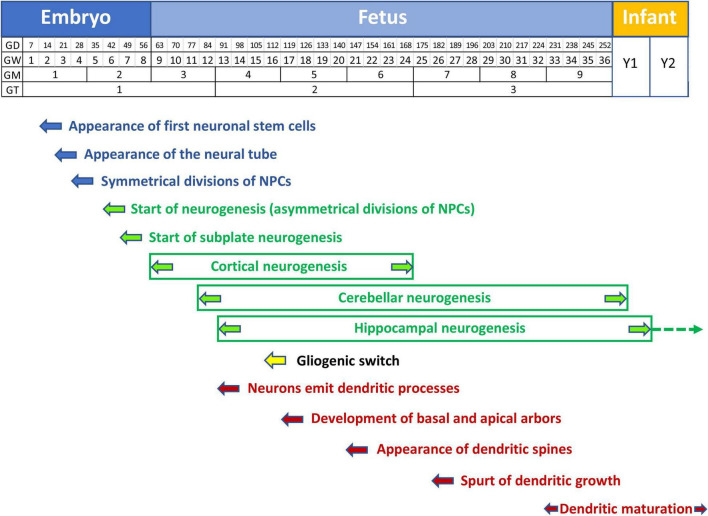
Timeline of neurogenesis, gliogenesis, and neuron maturation in the human brain. Neural stem cells appear at GD14. Symmetrical divisions of NPCs that start at GD28 gradually shift at GD42 to neuron-producing asymmetrical divisions. Cortical neurogenesis starts at GW9 and is completed at GW24-25. Cerebellar and hippocampal neurogenesis continue beyond birth. Cerebellar neurogenesis stops at month 5. Hippocampal neurogenesis is largely completed by the first year but continues very slowly throughout life (dashed arrow). Starting from GW16 neuron-producing divisions are gradually replaced by glia-producing divisions (gliogenic switch). Gliogenesis extends beyond birth (yellow arrow). Neurons emit dendritic processes starting from GW13, but the dendritic spurt takes place much later at GW26, associated with the appearance of dendritic spines. Dendritic maturation begins shortly before birth (GW34) and is largely completed by the second postnatal year. Note that graphic representation of the postnatal time is not to scale with embryonic and fetal periods. Abbreviations: GD, gestation day; GM, gestation month; GT, gestation trimester; GW, gestation week; and Y, year.

From the end of gastrulation through approximately E42 in humans, the neuroepithelial proliferative cells of the VZ (NPCs) constitute a homogeneous pseudo-stratified epithelium. These cells have radial processes and divide “symmetrically” producing two identical NPCs (Bystron et al., 2008; Figure 1Aa). Various rounds of symmetrical cell division augment the size of the NPC pool and cause a surface expansion of the cerebral cortex. The NPCs generate subsequently radial glial cells (RGC) which share some molecular characteristics with earlier NPCs (Bystron et al., 2008). RGCs undergo “asymmetrical” divisions thereby producing one progenitor and one neuron (Figure 1Ab). The former remains in the proliferative niche, whereas the latter migrates to its final location in the developing brain. In the case of cortical neurogenesis, the first neurons that abandon the proliferative zone form a structure called the preplate (PP; Figure 1Ba), a largely transient structure that comprises various cell types, most of which are destined to die (Bystron et al., 2008). Once the PP is complete, the next wave of migrating neurons splits the PP into two regions, the marginal zone (MZ) and the subplate (SP), beginning to form a new region interposed between the MZ and the SP, the cortical plate (Figure 1Bb,c), which will become the cortex (Figure 1Bd). The MZ and the SP are two transient laminar compartments populated by diverse cell types that have a major role in the development of the cortex but that are largely eliminated by the end of the fetal period (Allendoerfer and Shatz, 1994). The MZ contains an important class of cells, the Cajal–Retzius cells, that control the positioning of neurons into the correct layers of cortex. It will become layer I of the mature cortex (Figure 1Bd). The SP contains multipolar neurons that play a functional role in setting up connections between cortex and thalamus during development. The SP in humans reaches its maximum thickness roughly two-thirds of the way through gestation (Bystron et al., 2008). Its size then gradually decreases, leaving only a thin layer with scattered cells in the white matter in the late fetal period (Figure 1Bd). At the beginning of cortical development neurons migrate through a process called somal translocation. During later stages this process is no longer possible, due to brain growth, and so neurons migrate to the cortex along the shafts of RGCs (Figure 1Ab). Cohorts of postmitotic neurons follow radial glial scaffolding to form arrays of minicolumns. The larger the number of columns, the larger the cortical surface. Neurons that arrive first settle in the prospective layer VI, while later migrating neurons settle to successively more superficial layers. This pattern of migration is called inside-out and causes an expansion in cortical thickness (Figure 1C). It has been estimated that in humans neurogenesis of the cells that will be found in the SP starts at day 47 post-conception and that those that form cortical layer VI are produced starting at day 57 (Clancy et al., 2001). When does cortical neurogenesis stop? Malik et al. (2013) addressed this issue based on the fact that a transient neural progenitor population called intermediate progenitor cells (Figure 1Ac), which are exclusively neurogenic and generate glutamatergic neurons, express Tbr2 but not Sox2 (that is expressed by radial glia). They found that the number of Tbr2-positive cells is very high at GW16–GW19, decreases to approximately one third at GW23–GW25, and becomes extremely small by GW26–GW28, to disappear thereafter. According to Malik et al. (2013) and a subsequent study (Kostović et al., 2019), the end of cortical neurogenesis can be placed at GW24–GW25 (Figure 2). While cortical neurogenesis is completed by the second trimester, cerebellar and hippocampal neurogenesis extends for a more prolonged period. Production of cerebellar granule cells starts at GW12 (ten Donkelaar et al., 2003) and continues as late as the fifth postnatal month (Abraham et al., 2001; Figure 2). In the hippocampal dentate gyrus (DG), production of granule cells begins at GW12–GW13, slowly continues during the first postnatal year (Seress et al., 2001; Rice and Barone, 2010), and, at a much slower rate, throughout life (Eriksson et al., 1998; Boldrini et al., 2009; Spalding et al., 2013; Moreno-Jiménez et al., 2021; Figure 2). In addition to the VZ/SVZ, a proliferative region exists in the ganglionic eminences of the ventral telencephalon. In rodents, this transient region is the source of inhibitory (GABAergic) interneurons that reach their final location through tangential migration in the dorsal telencephalon (Brazel et al., 2003; Figure 1D). In humans, however, inhibitory interneurons are also born in the VZ/SVZ of the dorsal telencephalon (see Rakic, 2009).

### Gliogenesis

During cortical development, neurons are generated first, followed by astrocytes, and then oligodendrocytes (see Sauvageot and Stiles, 2002; Lanjewar and Sloan, 2021 for a review). Glial cells make up at least 50% of brain cells (Rowitch and Kriegstein, 2010). Glial progenitors begin to be produced prenatally but the processes of proliferation, migration, differentiation, and maturation continue after birth. While much is known regarding the timing of these processes in rodents, scarce information is available regarding humans. In rodents, VZ neurogenesis begins at E12 and lessens by E17. Cells produced by the SVZ, which is by now the primary germinal zone, principally become glia in the period E17-postnatal (P) day 14. Astrocyte and oligodendrocyte generation peaks at P0–P2 and P14, respectively. The relative timing of these processes in humans is thought to reflect that seen in rodents (Figure 1E). In humans, NPCs give rise to neuronal restricted progenitors early in development, and to glial restricted progenitors only later. Around GW16–GW18, radial glia slowly begin to form astrocytes and oligodendrocytes, a process by which the same progenitor domain switches the developmental program from neuron production to astrocyte or oligodendrocyte production. The neurogenic to gliogenic cell fate transition of radial glia is called “gliogenic switch” (Figure 2). The gliogenic switch in cell fate is regulated by a combination of extrinsic, intrinsic, and epigenetic signals (Rowitch and Kriegstein, 2010). Gliogenesis may occur postnatally because astrocytes can be locally generated in the cortex (Ge et al., 2012) and oligodendrocyte precursor cells that are already resident in the gray matter can slowly produce oligodendrocytes (Rowitch and Kriegstein, 2010). Microglia cells that, unlike astrocytes and oligodendrocytes are of mesodermal origin (Rowitch and Kriegstein, 2010), invade the brain as early as GW5. Microglial migration and proliferation continue until around GW24 (see Lanjewar and Sloan, 2021).

### Neuron Maturation

Neurons that have settled in the cortex develop dendrites and axons to communicate with other neurons. Excitatory inputs are established mainly on dendritic spines, whereas inhibitory inputs contact non-spiny dendritic portions. In humans, dendritogenesis starts as early as GW13.5–GW15 and during GW17–GW25 the basic features of the apical and basal dendrites of cortical pyramidal neurons develop, with a spurt in growth at GW26–GW32 (Mrzljak et al., 1988, 1992; Figure 2). At GW27 fibers from the thalamus invade the cortical plate (Kostovic and Judas, 2010). It is noteworthy that the timeline of dendritic development may vary according to neuron location (Becker et al., 1984; Lu D. et al., 2013). For instance, the dendrites of layer V pyramidal neurons attain their maximum size earlier (4 months of age) than layer III neurons (2 years of age; Becker et al., 1984). The dendritic spurt of prefrontal cortex pyramidal neurons (GW26–GW32) is accompanied by the appearance of dendritic spines (Mrzljak et al., 1988, 1992; Figure 2). Dendritic spines in hippocampal pyramidal neurons occur at GW22–GW26 and the process is completed by the end of postnatal months 5–6 (Purpura, 1975; Lu D. et al., 2013).

## Spatiotemporal Characteristics of Neurodevelopmental Alterations in Down Syndrome

Information regarding the developing DS brain derives from fixed brain samples, sonographic and, more recently, MRI examinations. Evidence is mainly available for the last 2/3 of the second trimester. What happens before this remains obscure although it can be inferred that changes observed at later stages are the outcome of earlier occurring pathogenic events. Below we review our knowledge regarding brain development in fetuses with DS.

### The Brain of Down Syndrome Fetuses Is Hypotrophic

The fetal DS brain is reduced in weight, volume, and linear size (see Stagni et al., 2018). Size defects have been detected as early as GW 14.7, involve forebrain structures and the cerebellum, and are in the range between −10 and −30% vs. control brains (see Stagni et al., 2018). Recent MRI studies in the living fetus (Patkee et al., 2020; Tarui et al., 2020) have consented a quantification of the dimensions of the fetal DS brain at different time points. Fetuses with DS (GW21–GW35) have a reduced volume of the cerebellar hemispheres, whole cerebellum, cortical plate, and subcortical parenchymal volume compared to controls and the difference increases with gestation (Tarui et al., 2020). Likewise, Patkee et al. (2020) found a reduction in whole brain and cerebellar volume in the second and third trimester. These multiple approaches provide unequivocal proof that brain hypotrophy is a typical phenotype of DS starting from early fetal life stages, retained at later fetal stages, and postnatally (see Stagni et al., 2018).

### Early Hypocellularity in the Down Syndrome Fetus

Brain hypotrophy in fetuses with DS might be due to a reduction in the number of cells forming the brain and/or a reduction in the extension of their dendritic (and axonal) processes. Considering that brain hypotrophy has been detected well before the spurt of dendritic growth (see Figure 2), it seems very likely that it is due (or mainly due) to a lack of cellular elements. Indeed, a reduction in cellularity has been documented in several brain regions of DS fetuses in the period GW17–GW21 (earlier evidence is missing). These regions include the whole cerebrum (Larsen et al., 2008), the hippocampus, DG, presubiculum and entorhinal cortex (Guidi et al., 2008), the subiculum (Stagni et al., 2019a), the cortex of the inferior temporal gyrus and fusiform gyrus (Guidi et al., 2018), and some thalamic nuclei (Stagni et al., 2020). This reduction ranges between -22 and -35%, indicating a large deficiency in the number of brain cells. In addition, the fetal cortex has layers that are disorganized in comparison with the normal brain (Takashima et al., 1981; Becker et al., 1991; Golden and Hyman, 1994; Engidawork and Lubec, 2003; Guidi et al., 2018), which suggests impairment in circuit formation.

### Neurons Are a Missing Population in the Fetal Down Syndrome Brain

Regarding hypocellularity in the fetal DS brain, the question arises whether the missing population is represented by neurons, glial cells, or both. Studies in fetuses with DS show that at GW17–GW21, in the DG, hippocampus, presubiculum, entorhinal cortex, subiculum, inferior temporal gyrus, and fusiform gyrus (i) the majority of cells (∼75–95%) are neurons (NeuN+ cells), which is fully consistent with the delayed timing of gliogenesis in comparison with neurogenesis and, (ii) in DS fetuses the missing population is represented by neurons but not by astrocytes (Guidi et al., 2008, 2018; Stagni et al., 2019a). In line with this histological evidence, analysis of protein expression in fetuses with DS (GW 19.6 ± 2.0) shows a reduction in beta-tubulin (Engidawork et al., 2003), a protein that is specifically expressed by neurons. Taken together, these studies show that a deficit in neuron number underlies the hypocellularity that characterizes the fetal DS brain.

### Astrocytes Are Not a Missing Population in the Fetal Down Syndrome Brain

While at GW17–GW22 neurons were found to be reduced in number, this was not the case for astrocytes, the absolute number of which did not differ between DS and control fetuses (Guidi et al., 2008, 2011, 2018; Stagni et al., 2019a). Consistent with the timing of gliogenesis, that peaks at later stages, at GW17–GW22 astrocytes (GFAP+ cells) were only ∼5–18% of total cells. Zdaniuk et al. (2011) found that at GW18–GW20 DS fetuses may even have a larger number of astrocytes in comparison with controls. A larger expression of the glioprogenitor marker GFAP, accompanied by a reduction in the level of the neuroprogenitor marker Paired box 6 (PAX6), was found in the DS fetal frontal cortex at GW14 and GW21 (Lu et al., 2011). Likewise, in the VZ/SVZ of DS fetuses (GW18) there is a reduced percentage of cells expressing neuronal markers and a higher percentage of cells expressing the glial markers GFAP and oligodendrocyte transcription factor 2 (OLIG2; Lu et al., 2012). An increase in the number of GFAP- or S100B-positive cells has been additionally documented in cultures of trisomic human induced pluripotent stem cells (hiPSCs; Briggs et al., 2013; Chen et al., 2014; Hibaoui et al., 2014). Thus, astrogliogenesis is not compromised and may even be enhanced in the fetal DS brain. Studies regarding astrocytes after mid-gestation are lacking. In infants with DS, astrocytes exhibit a deficit of interlaminar processes, suggesting impairment in their maturation (Colombo et al., 2005). However, the propensity for astrogliogenesis is not a positive event in DS because astrocyte functioning is impaired, which may negatively affect neuronal function (see Ponroy Bally and Murai, 2021).

### Oligodendrocytes Are Not a Missing Population in the Fetal Down Syndrome Brain

Lu et al. (2012) found that in the VZ/SVZ of DS fetuses (GW18) there is a larger percentage of cells expressing OLIG2 (putatively oligodendrocyte precursor cells). The studies by Guidi et al. (2008, 2018) and Stagni et al. (2019a) show that cells that were neither NeuN- nor GFAP-positive represented a small fraction of total cells and that their number was similar (or even higher) in DS vs. control fetuses. These cells may include the precursors of oligodendrocytes and astrocytes as well as oligodendrocytes, suggesting no impairment in their generation. Trisomic NPCs obtained from hiPSCs give rise to fewer neurons but more astrocytes as well as oligodendrocytes (Hibaoui et al., 2014), confirming no impairment or even enhancement of oligogliogenesis. Transcriptome analysis shows that genes associated with oligodendrocyte progenitor cells gradually increase in the DS brain in comparison with controls from early mid-gestation to middle-adulthood (Olmos-Serrano et al., 2016), which also suggests an increase in oligogliogenesis. Contrariwise, genes associated with myelinating oligodendrocytes are expressed at lower levels from birth through adulthood, suggesting impairment in oligodendrocyte maturation (Olmos-Serrano et al., 2016). Moreover, the expression of the myelin components myelin basic protein and myelin associated glycoprotein are reduced starting from mid-gestation and the early neonatal period, respectively (Olmos-Serrano et al., 2016). This is consistent with the myelination impairment seen in individuals with DS from early postnatal life stages into adulthood (Wisniewski and Schmidt-Sidor, 1989; Becker et al., 1991; Koo et al., 1992; Abraham et al., 2011). Thus, although oligogliogenesis is not impaired in DS, impaired oligodendrocyte maturation prevents proper oligodendrocyte functioning.

### Proliferation Potency Impairment in the Fetal Down Syndrome Brain

In human beings, proliferation potency of NPCs can be indirectly estimated by quantifying the pool of actively dividing cells. This can be done in fixed brain sections by using immunohistochemistry for endogenous proteins expressed during the cell cycle, such as Ki-67, which is expressed during most of the cell cycle, Cyclin A, which is expressed during the S-phase, and phospho-hystone H3, which is expressed during the M-phase. Very few studies have evaluated the number of proliferating cells in the fetal DS brain. The available evidence shows that DS fetuses have a reduced number of proliferating cells in the VZ/SVZ of the frontal cortex (GW18; Lu et al., 2012), the ventricular germinal matrix of the inferior horn of the lateral ventricle, VZ/SVZ of the hippocampus, parahippocampal gyrus, and subiculum, various germinal zones of the DG, the external granular layer of the cerebellum, and VZ/SVZ of the third ventricle (GW17–GW23; Contestabile et al., 2007; Guidi et al., 2008; Stagni et al., 2019a,^2020^). In addition, the number of proliferating cells is reduced in a region of the cerebellum that is the remnant of the cerebellar VZ (Guidi et al., 2011). Finally, fetuses with DS at GW16–GW24 have a reduced number of SOX2+ cells (radial glia progenitors) in the VZ/SVZ of the frontal lobe (Baburamani et al., 2019). All these data suggest that in the fetal DS brain NPCs proliferate at a slower rate compared to controls. Since direct information on the length of the cell cycle cannot be obtained in human beings, some investigators have measured the length of the cell cycle in the Ts65Dn mouse model of DS, providing direct evidence of cell cycle elongation in the embryonic VZ (Chakrabarti et al., 2007) and in germinal layers of the cerebellum of neonate mice (Contestabile et al., 2009). The reduction in proliferation potency seen in the fetal brain is confirmed by evidence in cultures of NPCs derived from DS-hiPSCs showing that trisomic NPCs proliferate at a slower rate in comparison with controls, give rise to fewer neurons and exhibit reduced levels of genes involved in neurogenesis (Chen et al., 2014; Hibaoui et al., 2014; Murray et al., 2015; Sobol et al., 2019). Very recent evidence shows that DS-hiPSC-derived cerebral organoids present defects that are very similar to those of the fetal DS brain, such as volume reduction, reduced number of proliferating cells in VZ-like regions, reduced number of neuronal progenitors (SOX2+ cells), and no change in the expression of apoptotic markers (Tang et al., 2021). Taken together, data reported in this and preceding sections (“Neurons Are a Missing Population in the Fetal Down Syndrome Brain,” “Astrocytes Are Not a Missing Population in the Fetal Down Syndrome Brain,” and “Oligodendrocytes Are Not a Missing Population in the Fetal Down Syndrome Brain”) strongly suggest that brain hypotrophy in DS is due to a paucity of neurons that is caused by neurogenesis impairment.

### Apoptotic Cell Death May Contribute to Reduce Neuron Number in the Fetal Down Syndrome Brain

The process of neurogenesis is accompanied by naturally occurring cell death (apoptosis). This physiological process eliminates approximately 50% of the new neurons, thereby shaping future neural circuits. Conflicting results are available regarding apoptosis in the fetal DS brain. There is evidence of no change in apoptosis in the cerebellum, hippocampus, and parahippocampal gyrus at GW17–GW21, but of an increase in the VZ/SVZ and DG (Guidi et al., 2008, 2011). Likewise, an apoptosis increase was found in the VZ/SVZ of DS fetuses at GW18 (Lu et al., 2011). An increase in apoptosis was also detected in cultures of trisomic hiPSC-derived NPCs (Hibaoui et al., 2014), although another study found no changes (Sobol et al., 2019). Since, at least in the second trimester, the number of cells undergoing apoptosis in the brain is very low both in euploid and DS fetuses (Abraham et al., 2001; Guidi et al., 2008, 2011), the quantitative relevance of apoptosis in reducing the final neuron number in DS remains to be established.

### Spatiotemporal Characteristics of Neurogenesis Alterations in Down Syndrome

The studies reviewed above show that in the fetal DS brain proliferation potency is impaired at several locations along the rostro-caudal axis of the VZ/SVZ of the cerebrum, in the VZ/SVZ of the III ventricle and in various neurogenic niches of the DG and cerebellum. Thus, proliferation impairment appears to have a spatially large distribution.

Due to the lack of fetal brain samples during the first trimester, it cannot be established whether at the beginning of neurogenesis (GW6) DS fetuses have the same asset of neural stem cells as controls. Even if this were the case, the hypocellularity found at GW17 and the reduced number of dividing cells around this age indicate that at some point after the onset of neurogenesis the renewal of NPCs must begin to slow down. Differences in the expression of genes involved in neuron development and differentiation have been detected in the cerebral cortex of DS fetuses as early as GW14 (Olmos-Serrano et al., 2016), which suggests that neurogenesis defects are already present at this time. It must be recalled that symmetrical cell divisions taking place at GW4-5 provide a means for cortical surface expansion, due to an increase in the number of founder cells that give rise to radial cortical columns, whereas asymmetrical divisions (from GW6 on) provide the means to increase cortical thickness within radial columns without a change in cortical surface area (Rakic, 2003, 2009). Linear measurements of the fetal DS brain revealed a reduction in the fronto-occipital (Schmidt-Sidor et al., 1990; Guihard-Costa et al., 2006; Patkee et al., 2020) and biparietal diameters (Guihard-Costa et al., 2006), and a reduced length of the frontal lobe (Bahado-Singh et al., 1992), features that are suggestive of a reduced cortical expansion. In addition, fetuses with DS have lower average brain sulcal depths and gyrification indexes than control fetuses (Yun et al., 2021) which also suggests a reduced cortical expansion, because cerebral convolutions are formed in parallel with an increase in cortical surface without a comparable increase in cortical thickness (Rakic, 2004). On the other hand, during the second trimester fetuses with DS exhibit a notable reduction in cortical thickness (Golden and Hyman, 1994; Guidi et al., 2018) and a reduction in the number of cells expressing the radial glia marker SOX2 and of radial glia processes (Hutton and Pevny, 2011; Guidi et al., 2018), which suggests impairment in asymmetrical cell divisions. Based on this evidence, it seems conceivable that in DS fetuses there is a reduced rate of symmetrical cell divisions, during early neurogenesis in the first trimester, followed later by a reduced rate of asymmetrical cell divisions. While the second possibility is substantiated by the reduction in proliferation potency seen during the second trimester of gestation, the first possibility is merely speculative, due to a lack of direct information at earlier ages.

### Impaired Neuron Maturation in the Developing Down Syndrome Brain

Very few studies have examined dendritic development in DS. Takashima et al. examined neurons from the visual cortex of fetuses, neonates, and adults with DS (Takashima et al., 1981) and found a reduction in the length of the basal dendrites in infants who were older than 4 months. Becker et al. (1986) found that in infants younger than 6 months branching and length of apical and basal dendrites of visual cortex neurons were larger than in controls but that they were reduced after 2 years of age. Prinz et al. (1997) showed that a 3-month-old infant with DS had cortical interneurons with a higher number of branching points but reduced dendritic areas. A reduction in spine density and aberrant spine shape in neocortical and hippocampal neurons was detected in fetuses and children with DS. Takashima et al. (1981) found that at GW20 and GW23 pyramidal neurons in the visual cortex had long, thin spines. At GW40 and at 3 months spines were shorter, i.e., more mature. No differences in spine density were seen between DS and controls at these ages. At and after 4 months of age, however, dendritic spine density was reduced. Moreover, while in controls spine density increased up to 15 years of age this did not occur in children with DS (Takashima et al., 1994). Unusually long and tortuous (i.e., immature) dendritic spines were observed in the motor cortex of a 19-month-old child with DS (Marin-Padilla, 1976). Alteration in spine structure and decreased spine density in hippocampal neurons were also observed in two children with DS aged 8 and 9 months (Purpura, 1975). This evidence shows that defects in dendritic maturation appear in early infancy.

There is a paucity of studies that have characterized the dendritic pattern in mouse models of DS. Early evidence showed that cortical neurons od Ts65Dn mice exhibit dendritic defects that parallel those found in humans (see Benavides-Piccione et al., 2004). Further studies found reduced density of dendritic spines and dendritic hypotrophy in hippocampal granule neurons of adult Ts65Dn mice (Belichenko et al., 2004; Guidi et al., 2013; Dang et al., 2014; Stagni et al., 2015b) and reduced spine density in granule neurons of Ts1Cje mice (Belichenko et al., 2007). Defects in dendritic complexity and dendritic spine density of hippocampal granule neurons were also detected in Ts65Dn mice aged 15 days (Stagni et al., 2017a,2019b; Emili et al., 2020). Recent evidence shows that granule neurons of Ts65Dn mice already exhibit dendritic hypotrophy and spine shape (but not density) alterations at postnatal day 8 (Uguagliati et al., 2021). The presence of dendritic branching defects has been additionally found in neocortical pyramidal neurons of Ts65Dn pups aged 2 days (Uguagliati et al., 2022). Taken together the latter two studies are in line with the early presence of dendritic alterations seen in infants with DS.

While neurogenesis (and other) defects of DS may be shared with some other types of ID, such as fragile X syndrome (Bardoni et al., 2017), fetal alcohol spectrum disorder (Miranda, 2012), and autism spectrum disorders (Bicker et al., 2021), dendritic alterations are shared with virtually all mental disorders (Dierssen and Ramakers, 2006; Quach et al., 2021; Granato and Merighi, 2022). It has been suggested that cognition defects in different types of ID may be underpinned by alterations of different dendritic domains (Granato and Merighi, 2022). In this connection it is interesting to note that dendritic branching defects in Ts65Dn pups mainly involve the basal domain shortly after birth (postnatal day 2) and the apical domain slightly later (postnatal day 8) suggesting a relationship between age, affected dendritic compartment (Uguagliati et al., 2022) and, possibly, cognitive impairment in DS.

## Genes Responsible for Neurogenesis Impairment in Down Syndrome

The preceding sections have shown that the fetal DS brain exhibits a reduced number of proliferating NPCs. Were the fate of their progeny unaltered, the outcome would be a proportional reduction in the number of neurons and glial cells. However, only the number of neurons is reduced in DS, while that of astrocytes and oligodendrocytes is unchanged or increased. This implies deregulation of the genetic mechanisms that control proliferation potency (cell cycle progression) as well as of those that control cell fate.

In addition to genes on Chr21, many genes throughout the genome are differentially expressed in the DS brain (Olmos-Serrano et al., 2016) and many genes are hypermethylated (i.e., their transcription is repressed; El Hajj et al., 2016), which highlights a potentially enormous complexity in neurogenesis regulation. Nonetheless, most of the investigations carried out so far have focused on those triplicated genes on Chr21 (and related pathways) that are thought to be important for the proliferation and fate of NPCs. Among candidate genes, Dual-specificity tyrosine phosphorylation-regulated kinase 1A (*DYRK1A*), amyloid beta precursor protein (*APP*), Regulator of calcineurin 1 (*RCAN1)* have been more widely investigated, although additional genes (described below) are emerging as potential candidates. For simplicity, we will describe the genes and mechanisms that impair (i) proliferation and (ii) neurogenesis (i.e., acquisition of a neuronal vs. an astrocytic phenotype) in two separate sections, although this distinction is somewhat forced because these processes may be intermingled.

### Mechanism of Neural Progenitor Cell Proliferation Impairment

**FIGURE 3:**
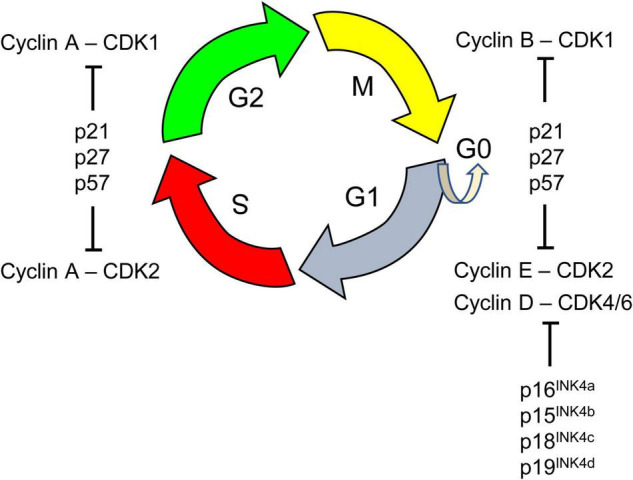
The cell cycle is a series of events that lead to cell division. It comprises four phases: the first gap phase called G1 during which cells prepare for DNA replication, the S phase of DNA synthesis, a second gap phase called G2, and the M phase of cell mitosis in which segregation of replicated chromosomes into two separate cells occurs. Cells in G1 can, before DNA replication, enter a resting state, called G0. Progression through the cell cycle is tightly regulated by cyclin-dependent kinases (CDK 1, 2, 4, 6). CDKs are serine/threonine protein kinases that phosphorylate key substrates for DNA synthesis and mitotic progression. CDKs interact with various positive and negative cell cycle regulators. Cyclins (A, B, D, E) are positive cell cycle regulators that represent the regulatory subunits of CDKs; their binding to CDKs allows inactive CDKs adopting an active configuration, driving transition phases. Different cyclins are required at different phases of the cell cycle. For instance, D-type cyclins bind to CDK4/6 forming the active cyclin D/CDK4/6 complex that is necessary for progression through the G1 phase of the cell cycle. The G1 phase is considered a critical window during which cells decide to proliferate, assume a reversible arrest (G0), or begin a path toward terminal differentiation or senescence. The activity of CDKs can be counteracted by the CDK inhibitory protein/Kinase inhibitory protein (Cip/Kip) family, that includes p21^CIP1^ (p21), p27^KIP1^ (p27), p57^KIP2^ (p57) and by proteins of the INK4 family. These negative cell cycle regulators inhibit cell cycle progression by binding to specific CDKs alone (INK4 family) or cyclin-CDK complexes (Cip/Kip family).

Evidence in fetuses and mouse models suggests that the reduced size of the pool of NPCs in the fetal DS brain is due to changes in cell cycle dynamics. The latter is regulated by (i) cyclin-dependent kinases (CDKs), (ii) their interactions with cyclins, and (iii) Cip and Kip inhibitors of CDK activity (Figure 3). As shown below, many of these regulatory mechanisms are disrupted in DS.

#### Dual-Specificity Tyrosine Phosphorylation-Regulated Kinase 1A

**FIGURE 4:**
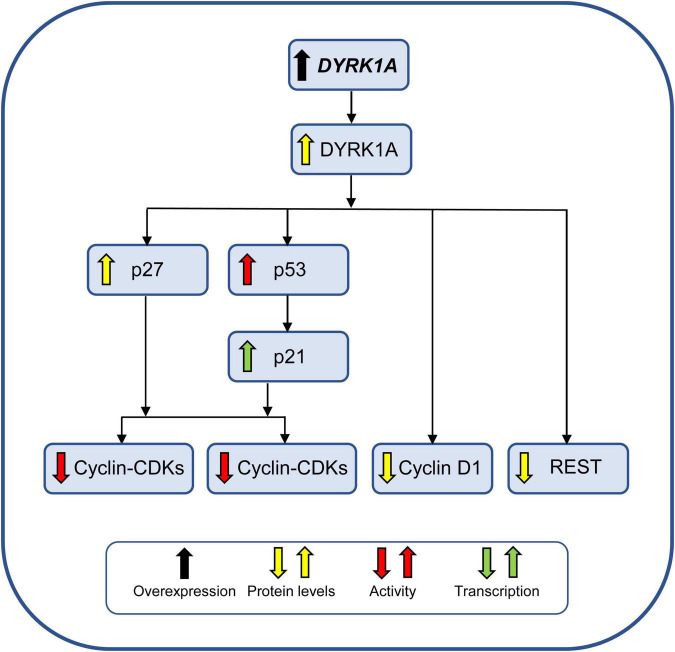
Effect of *DYRK1A* overexpression on NPC proliferation. DYRK1A impairs proliferation of NPCs by reducing the activity of different cyclin-CDKs and by reducing the levels of REST, a key regulator of pluripotency and neuronal differentiation. See text for further explanation. Abbreviations: DYRK1A, dual-specificity tyrosine phosphorylation-regulated kinase 1A.

*DYRK1A* is the more intensively studied gene in DS (Atas-Ozcan et al., 2021) because it plays an important role in neurogenesis, is highly expressed during embryonic neurogenesis, and *Dyrk1a* transgenic mice exhibit brain alterations that are reminiscent of DS (Hammerle et al., 2003). DYRK1A is a kinase that phosphorylates a multitude of targets, including transcription factors. Regarding its expression in the fetal DS brain, *DYRK1A* resulted as being overexpressed (RNA) at GW15–GW37 (El Hajj et al., 2016) and GW20 (Guimera et al., 1999), and DYRK1A protein was overexpressed at GW23 (Park et al., 2010). However, no changes at the protein level were found at GW18–GW19 (Cheon et al., 2003a) or in infants aged 1–3 years, although DYRK1A was more widely expressed in DS adolescents and adults (Dowjat et al., 2007). These discrepancies prompt further investigations. There are four major mechanisms whereby overexpression of *DYRK1A* may impair NPC proliferation in DS (summarized in Figure 4).

1) Overexpression of DYRK1A impairs the cell cycle (Figure 3) by directly affecting the levels of negative and positive regulators of cell cycle progression. In particular, overexpression of DYRK1A increases the levels of the antiproliferative CDKs inhibitor p27^KIP1^ and promotes its stability by phosphorylating it on Ser(10) (Hammerle et al., 2011; Soppa et al., 2014). This action of DYRK1A on p27^KIP1^ in conjunction with its action on cyclin D1 (see below) inhibits cell cycle progression through the G1 phase, promotes cell cycle exit into G0 and subsequent premature neuronal differentiation. This effect can be prevented by normalization of DYRK1A activity with harmine (Mazur-Kolecka et al., 2012).
2) DYRK1A phosphorylates p53, resulting in the transcription of p53 target genes, including p21^CIP1^ (Park et al., 2010) which impairs G1/G0-S phase transition. Brains from embryonic *Dyrk1a* transgenic mice have high levels of phosphorylated p53, and p21^CIP1^, and reduced neuronal proliferation (Park et al., 2010). Increased levels of DYRK1A, p53, and p21^CIP1^ have been found in the frontal cortex of fetuses and adults with DS (Park et al., 2010), and increased levels of p21^CIP1^ have been found in the brains of fetuses with DS (Engidawork et al., 2001).
3) An additional mechanism consists in a cyclin D1-dependent precocious exit from the cell cycle and premature neuronal differentiation (Tejedor and Hammerle, 2011; Hindley and Philpott, 2012; Chen et al., 2013; Najas et al., 2015). DYRK1A phosphorylates cyclin D1 at Thr(286) (Chen et al., 2013) which allows for its nuclear export followed by degradation (Yabut et al., 2010; Soppa et al., 2014). Reduction of cyclin D1 nuclear levels causes an increase in G1 duration and precocious exit from the cell cycle (Chen et al., 2013). The validity of this working model is supported by evidence obtained in DS fibroblasts and the Ts65Dn model which show an extended G1 duration that can be reversed by DYRK1A inhibition or knockdown (Chen et al., 2013). Radial glia progenitors in the VZ of Ts65Dn embryos have reduced cyclin D1 levels and a lengthening of the G1 phase (Najas et al., 2015). These alterations curtail the number of neuron-producing divisions and, thus, impair neurogenesis. Normalization of *Dyrk1a* dosage restores cyclin D1 levels and the number of cortical neurons (Najas et al., 2015).
4) The repressor element-1 silencing transcription factor (REST) modulates the expression of genes encoding important neuronal functions and is a key regulator of target genes for the transition from pluripotent embryonic stem cells to NPCs and, subsequently, to mature neurons (see Canzonetta et al., 2008). REST transcriptional levels are reduced in neural stem cells and NPCs from the cortex of fetuses with DS (Bahn et al., 2002; El Hajj et al., 2016) and DS hiPSC-derived NPCs (Hibaoui et al., 2014). REST transcriptional levels have also been found to be reduced in transchromosomic mouse embryonic stem cells (containing an extra copy of chromosome 21) with concomitantly reduced expression of two key pluripotency regulators, Nanog and Sox2, resulting in aberrantly premature expression of transcription factors driving early endodermal and mesodermal differentiation (Canzonetta et al., 2008). Dyrk1a dosage imbalance in embryonic stem cells was found to perturb REST expression, with both over- and under expression of Dyrk1a resulting in REST suppression. This evidence suggested that REST dysregulation in trisomic cells was mediated by overexpression of Dyrk1a. Importantly, partial knockdown of Dyrk1a increased the reduced expression of Nanog and Sox2 (Canzonetta et al., 2008), strongly suggesting that DYRK1A-mediated deregulation of REST in DS plays a role in the alterations of pluripotency and embryonic stem cell fate.

#### Amyloid Beta Precursor Protein

**FIGURE 5:**
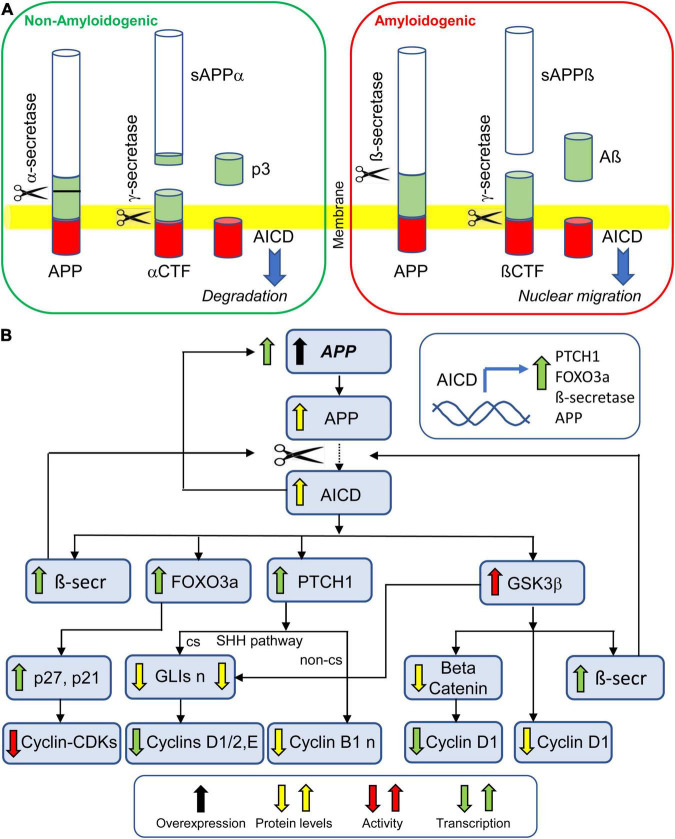
Effect of *APP* overexpression on NPC proliferation. **(A)** Proteolytic processing of APP by non-amyloidogenic pathway and amyloidogenic pathway. Both pathways give origin to AICD. While AICD produced by non-amyloidogenic processing undergoes degradation, AICD produced by amyloidogenic processing translocates to the nucleus and promotes transcription of various genes (inset in **B**). **(B)** APP overexpression leads to excessive levels of AICD. The AICD promoted transcription of PTCH1 leads to reduced transcription of cyclin D1 (through the SHH canonical pathway) and reduced levels of cyclin B1 (through the SHH non-canonical pathway). The AICD promoted transcription of FOXO3a promotes the transcription of p27 and p21, thereby inhibiting the activity of cyclin-CDKs. The AICD promoted transcription of ß-secretase enhances the amyloidogenic cleavage of APP. The AICD-mediated increase in GSK3ß activity causes a beta-catenin-mediated reduction in cyclin D1 transcription, a reduction in cyclin D1 levels, due to its degradation, and an increase in the transcription of ß-secretase, thereby enhancing the amyloidogenic cleavage of APP. Abbreviations: AICD, intracellular C-terminal domain; APP, amyloid beta precursor protein; αCTF, αcarbossi terminal fragment; ßCTF, ßcarbossi terminal fragment; ß-secr, ß-secretase; cs, canonical signaling; n, nuclear; non-cs, non-canonical signaling; sAPPα, soluble APPα; and sAPPß, soluble APPß.

Accumulating evidence suggests that *APP* plays a key role both in neurogenesis alterations in DS and development of AD-like pathology in adulthood (Coronel et al., 2019). APP is hydrolyzed by α-, ß-, and γ-secretase to generate various fragments (Figure 5A), including Aß peptides and the APP intracellular C-terminal domain (AICD). Neurons mainly contain the APP695 isoform which preferentially forms sAPPß, Aß, and AICD (Belyaev et al., 2010). In human embryonic kidney 293 cells, overexpression of *APP* inhibits cell proliferation and affects the expression of genes involved in G1/S checkpoint regulation, cell proliferation, and p53 signaling (Wu et al., 2016), suggesting that overexpression of *APP* during fetal life stages may contribute to the impairment of neurogenesis. In the fetal DS brain, APP was found to be increased in the temporal cortex (protein) at GW17-21 (Guidi et al., 2017), whole cortex (protein) at GW14 and GW21 (Lu et al., 2011), whole cortex (RNA) at GW15-37 (El Hajj et al., 2016), and whole brain (protein and RNA) at GW19 (Tanzi et al., 1987, 1988). A single study detected no changes in APP protein levels in the fetal DS brain at GW18-19 (Cheon et al., 2003b). Higher APP levels were detected in DS hiPSCs generated from second trimester amniotic fluid (Lu H. E. et al., 2013) and cultures of DS-fetuses-derived cortical neurons (Busciglio et al., 2002). Moreover, high levels of various APP derivatives were detected in the fetal DS brain (Takashima et al., 1994; Teller et al., 1996; Russo et al., 2001). Regarding the mechanisms whereby excessive APP levels impair NPC proliferation, AICD is very likely a major effector because, as detailed below, AICD overexpression impairs both Sonic Hedgehog (SHH) signaling and Glycogen synthase kinase-3β (GSK3β) activity, both of which are involved in neurogenesis (Figure 5B).

1) AICD and the SHH pathway. Following interaction with Fe65, AICD translocates into the nucleus and promotes the transcription of various genes (see Nalivaeva and Turner, 2013; Coronel et al., 2019), including the gene encoding the transmembrane receptor PATCHED 1 (PTCH1; Trazzi et al., 2011; see inset in Figure 5B). Consistently with increased APP/AICD levels, PTCH1 is overexpressed in fetuses with DS and in Ts65Dn mice (Trazzi et al., 2011). PTCH1 is an SHH receptor that keeps the mitogenic SHH pathway repressed by inhibiting the transmembrane protein Smoothened (SMO), the activator of the SHH pathway. Canonical SHH signaling takes place when SHH binds and inactivates PTCH1 (Carballo et al., 2018). Once PTCH1 is inhibited, SMO is activated and initiates the SHH downstream signaling cascade. This results in the translocation of GLI proteins to the nucleus. Once activated, GLIs (GLI1, GLI2, and GLI3) bind to GLI-promoters and activate/inhibit gene transcription. These genes include *cyclin D1*, *cyclin D2*, and *cyclin E* (Kenney and Rowitch, 2000; Cayuso et al., 2006), the expression of which is enhanced following SHH pathway activation. Thus, reduced SHH pathway activation, due to excessive PTCH1 levels, causes a reduction in *cyclin D1*, *cyclin D2*, and *cyclin E* transcription (Figure 5B). It is worth mentioning that GSK3β belongs to the complex that prevents GLI migration into the nucleus (Pan et al., 2006), thereby potentiating the effects of excessive AICD levels on these cyclins. Non-canonical SHH signaling may take place independently of SMO; binding of SHH to PTCH1 disrupts its interaction with Cyclin B1, allowing cyclin B1 to localize to the nucleus which leads to an increase in cell proliferation and survival (Barnes et al., 2001; Carballo et al., 2018). This effect may be hampered by excessive PTCH1 levels (Figure 5B). Cyclin B1 is the regulatory subunit of CDK1, the key controller of mitosis entry (Takizawa and Morgan, 2000). Accordingly, in the cerebellum of Ts65Dn mice cyclin B1 levels are reduced and there is a disproportionate number of cells in G2 and a prolonged G2 phase (Contestabile et al., 2009). In summary, excessive PTCH1 expression results in reduced canonical and non-canonical SHH signaling, down regulation of cell-cycle components and, ultimately, proliferation impairment. This conclusion is substantiated by evidence that restoration of PTCH1 levels restores proliferation in trisomic NPCs (Trazzi et al., 2011) and that direct stimulation of SMO restores cerebellar granule cell proliferation in Ts65Dn pups (Roper et al., 2006). It should be noted that AICD promotes the transcription of the β-site APP cleaving enzyme 1 (ß-secretase, *BACE1*; Nalivaeva and Turner, 2013) which may result in enhanced production of APP derivatives, including AICD itself (Figure 5B).
2) AICD and GSK3β. GSK3β is a constitutively active kinase that is inhibited by an increase in phosphorylation at Ser(9). Reduced phosphorylation of GSK3β at Ser(9) was observed in NPCs from Ts65Dn mice (Trazzi et al., 2014), the hippocampus of Ts65Dn pups (Giacomini et al., 2015), and VZ of fetuses with DS (Trazzi et al., 2014). In NPCs derived from Ts65Dn mice, excessive AICD levels prevent GSK3β phosphorylation at Ser(9), thereby enhancing GSK3β activity (Trazzi et al., 2014). Since over activity of GSK3β impairs neurogenesis (and neuron migration; Kim and Snider, 2011), the APP-AICD-mediated increase in GSK3β activity is expected to impair proliferation in the fetal DS brain. This hypothesis is confirmed by evidence that inhibition of GSK3β restores proliferation of NPCs from the SVZ of Ts65Dn mice (Trazzi et al., 2014). Down regulation of cyclin D1 is most likely a key mechanism whereby GSK3β impairs proliferation (Figure 5B). This regulation may take place in a dual manner: (i) over-active GSK3β may directly increase cyclin D1 phosphorylation at Thr(286) and its nuclear export and degradation; (ii) over-active GSK3β increases beta-catenin phosphorylation and retains it in the cytoplasmic compartment. This prevents the action of beta-catenin that, when translocated into the nucleus, induces the expression of target genes, including cyclin Dl (Takahashi-Yanaga and Sasaguri, 2008; Figure 5B). It is of interest to note that GSK3β enhances ß-secretase expression through NF-kappaB signaling (Ly et al., 2013) which may result in enhanced production of APP derivatives, including AICD (Figure 5B) and amplification of the detrimental effects described above.
3) AICD and FOXO3a. Recent evidence in an AICD transgenic mouse model shows that AICD promotes the transcription of *Foxo3a*, a transcription factor that is expressed in NPCs and regulates neurogenesis and mitochondrial function (Jiang et al., 2020). While AICD-dependent regulation of FOXO3a inhibits hippocampal proliferation, suppresses neuronal stem cell differentiation, and increases cell death, functional loss of FOXO3a in NPCs of AICD transgenic mice rescues neurogenesis (Jiang et al., 2020). FOXO3a increases cell cycle inhibitor proteins p21 and p27 (Nho and Hergert, 2014), suggesting that an AICD-mediated increase in FOXO3a expression may concur to impair cell cycle progression in DS (Figure 5B).

#### Regulator of Calcineurin 1

**FIGURE 6:**
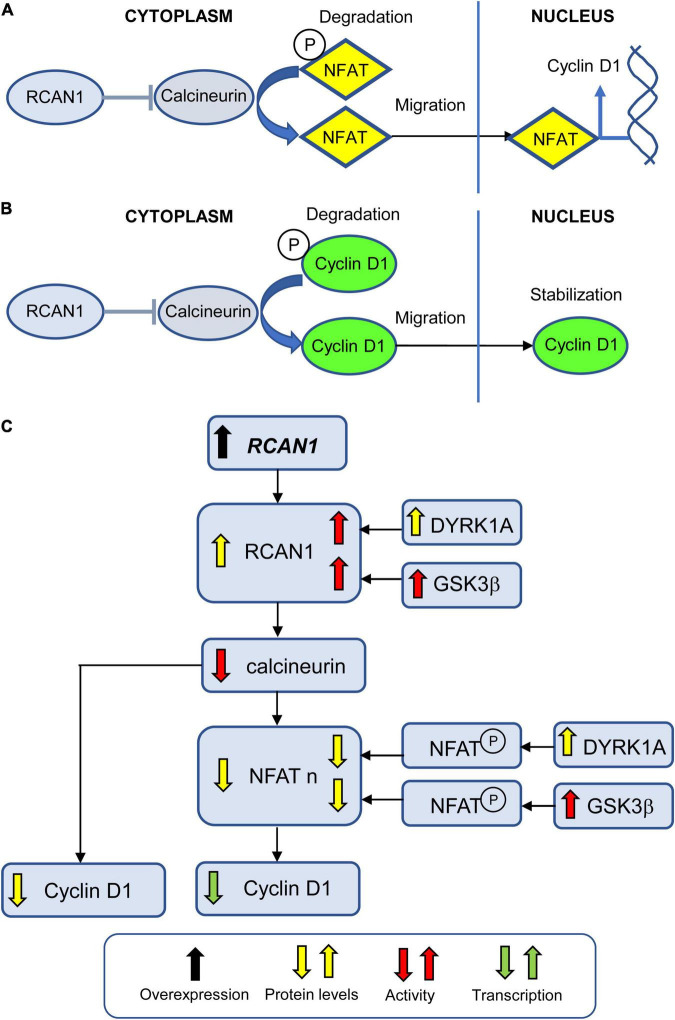
Effect of *RCAN1* overexpression on NPC proliferation. **(A)** Calcineurin dephosphorylates NFAT which allows its translocation to the nucleus where it promotes transcription of various genes, including cyclin D1. Phosphorylated NFAT remains in the cytoplasm where it undergoes degradation. RCAN1 inhibits calcineurin activity, thereby reducing NFAT nuclear translocation. **(B)** Calcineurin dephosphorylates cyclin D1 which allows its nuclear stabilization. Phosphorylated cyclin D1 remains in the cytoplasm where it undergoes degradation. RCAN1 inhibits calcineurin activity, thereby reducing cyclin D1 stabilization. **(C)** Excessive levels of RCAN1 increase the inhibition of calcineurin activity. This leads to reduced cyclin D1 protein levels due to its excessive degradation, and to reduced cyclin D1 transcription due to reduced NFAT translocation to the nucleus. DYRK1A and GSK3ß increase the activity of RCAN1, thereby increasing its inhibition on calcineurin. In addition, DYRK1A and GSK3ß enhance NFAT phosphorylation, thereby contributing to its degradation and reducing its nuclear levels. Abbreviations; RCAN1, regulator of calcineurin 1; NFAT, nuclear factor of activated T cell; and NFAT n, nuclear NFAT.

The Down syndrome critical region 1 (*DSCR1*), also named Regulator of calcineurin 1 (*RCAN1*), a member of a family of calcineurin binding proteins, is highly expressed in neuroproliferative zones during brain development and in various brain regions postnatally (Pritchard and Martin, 2013). *RCAN1* is overexpressed (RNA) in the fetal DS brain at GW20 (Guimera et al., 1999) and GW22 (Fuentes et al., 2000), in lymphoblastoid cell lines from children with DS (Granese et al., 2013), and in cultured amniocytes from fetuses with DS (Altug-Teber et al., 2007). Likewise, *Rcan1* is overexpressed in the embryonic brain of DS models (Kurabayashi and Sanada, 2013). RCAN1 interacts with calcineurin catalytic A subunit thereby inhibiting calcineurin-dependent signaling pathways. Calcineurin is a calcium and calmodulin-dependent serine/threonine protein phosphatase that activates the family of nuclear factor of activated T cell (NFATc or NFAT) transcription factors (the most studied substrates of calcineurin) through dephosphorylation (Figure 6A). In T cells, activated NFAT then translocates into the nucleus, where it upregulates the expression of Interleukin 2, which, in turn, stimulates growth and differentiation of T cells. In addition to T cells, NFATs are present in a variety of cells, including neurons and astrocytes. Inhibition of NFAT activation in NPCs from the SVZ reduces the percentage of cells in G0/1 and causes cell cycle elongation (Serrano-Perez et al., 2015). Human *RCAN1* transgenic mice, in which overexpression of RCAN1 was close to the level of overexpression observed in DS, exhibit defects in adult hippocampal neurogenesis and acquisition of a neuronal phenotype similar to those of DS (Martin et al., 2012). Taken together, these data suggest that RCAN1-dependent inhibition of calcineurin in the DS brain may maintain NFAT in its phosphorylated state, preventing its translocation to the nucleus and its pro-proliferative effects. This idea is strengthened by evidence of hyperphosphorylated NFATc4 in the fetal DS brain at GW20 (Arron et al., 2006). Regarding the mechanisms, there is evidence that dephosphorylation of NFAT by calcineurin promotes transcription of factors that promote proliferation, including cyclin D1 (Masaki and Shimada, 2022; Figure 6A). In addition, calcineurin exerts its phosphatase activity directly on cyclin D1, dephosphorylating it at T(286) (Goshima et al., 2019), thereby inhibiting its degradation (Figure 6B). An inhibitor of calcineurin (CN585) decreases cyclin D1 expression and delays G1-S progression (Goshima et al., 2019). Taken together these data suggest that overexpression of RCAN1 in DS hampers cell cycle progression by inhibiting calcineurin phosphatase activity which (i) reduces the NFAT-mediated activation of genes that favor cell cycle progression (including cyclin D1) and (ii) increases cyclin D1 degradation (Figures 6A,B).

#### Interactions Between Dual-Specificity Tyrosine Phosphorylation-Regulated Kinase 1A, Regulator of Calcineurin 1, and Glycogen Synthase Kinase-3β

Dual specificity tyrosine-phosphorylation-regulated kinase 1A and RCAN1 can act synergistically to control NFAT phosphorylation. DYRK1A phosphorylates RCAN1 at Ser(112) and Thr(192) residues (Jung et al., 2011). Phosphorylation of Thr(192) enhances the ability of RCAN1 to inhibit calcineurin, leading to reduced NFAT transcriptional activity (Jung et al., 2011; Figure 6C). DYRK1A can also reduce NFAT transcriptional activity through direct phosphorylation of NFAT (Arron et al., 2006; Figure 6C). In the mouse embryonic cortex, inhibition of NFAT activity, *via* increased levels of DYRK1A and RCAN1, causes a delay in cell cycle exit and neuronal differentiation of NPCs, and alteration of the laminar positioning of cortical neurons (Kurabayashi and Sanada, 2013). Consistently with this evidence, the delayed neuronal differentiation of progenitors in Ts1Cje is ameliorated by counteracting the dysregulated DYRK1A/RCAN1/NFAT pathway, either by reducing the expression of DYRK1A/RCAN1 or by activating NFAT (Kurabayashi and Sanada, 2013). RCAN1 activity is also modulated by GSK3β. Phosphorylation of RCAN1 at Ser(112) primes RCAN1 for GSK3β-mediated phosphorylation at Ser(108), which contributes to increasing RCAN1 activity. In addition, GSK3β phosphorylates NFAT proteins in the nucleus, resulting in their inactivation and export (Beals et al., 1997; Figure 6C).

### Mechanisms Impairing Neurogenesis and Favoring Gliogenesis in Down Syndrome

Differentiation of NPCs into either neurons or glia is regulated by the expression of proneural and progliogenic signals, respectively. Thus, triplicated genes that modify the expression of proneurogenic factors, such as Neurogenin1 (NGN1), Neurgenin2 (NGN2), Neurogenic differentiation factor1 (NEUROD), and mammalian achaete scute homolog-1 (MASH1) are likely to be strongly involved in the process of neurogenesis. The Janus kinase-signal transducer and activator of transcription (JAK-STAT) pathway plays a key role in gliogenesis (Bonni et al., 1997; Lee et al., 2016). Therefore, triplicated genes activating this pathway are likely to increase gliogenesis in DS.

#### Oligodendrocyte Transcription Factor 2

**FIGURE 7:**
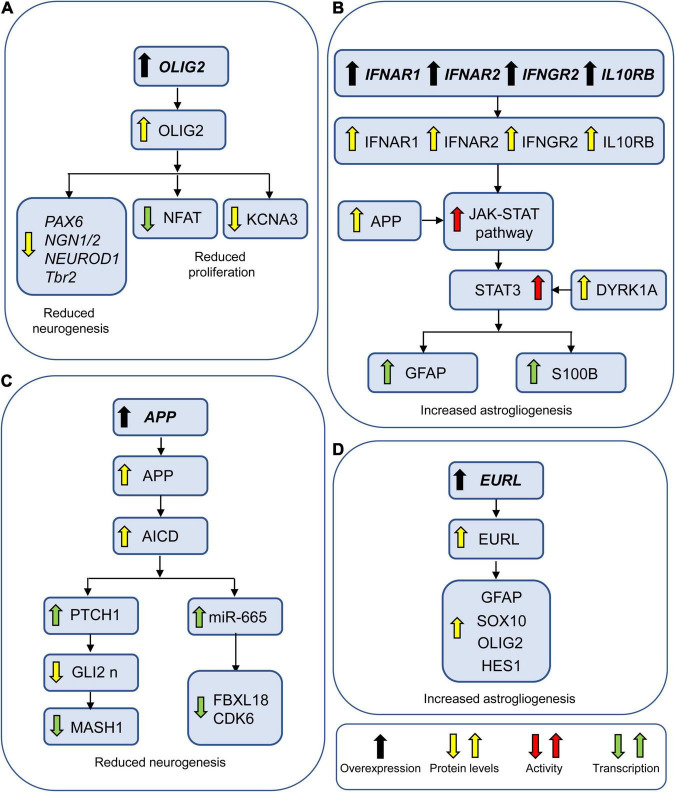
Genes involved in neurogenesis and gliogenesis alterations in DS. **(A)** *OLIG2* may reduce neurogenesis by reducing transcription of proneural factors. It may additionally impair NPC proliferation by reducing the expression of NFAT and the protein levels of the potassium channel KCNA3. **(B)** *IFNAR1*, *IFNAR2*, and *IFNGR2* may increase astrogliogenesis by activating the JAK-STAT pathway. APP and DYRK1A potentiate this effect by enhancing the activation of JAK-STAT pathway and STAT3, respectively. **(C)** AICD may reduce neurogenesis by causing overexpression of PTCH1 and miR-655. **(D)** EURL may favor astrogliogenesis by upregulating astroglial factors. Abbreviations: APP, amyloid beta precursor protein; EURL, early undifferentiated retina and lens; GLI2 n, nuclear GLI2; and OLIG2, oligodendrocyte transcription factor 2.

The oligodendrocyte transcription factor 1 (*OLIG1*) and *OLIG2* are both located on HSA21. They are thus named because of their key function in oligodendrocyte development. OLIG2 is fundamental for oligodendrogenesis and generation of motor neurons in the spinal cord (Lu et al., 2002). In the frontal cortex of fetuses with DS, *OLIG2* is overexpressed at GW14 and GW18, in parallel with proliferation reduction (Lu et al., 2012). OLIG2 overexpression is accompanied by reduced expression of the neural progenitor marker PAX6 and increased expression of GFAP (Lu et al., 2012). In transgenic mice with *Olig2* overexpression in nestin-expressing neural stem/progenitors, cells exhibit impairment in proliferation, precocious cell cycle exit, massive cell death, downregulation of proneural and neuronal differentiation genes, including *Ngn1*, *Ngn2*, and *Pax6*, as well as of *Nfatc4*, and a defect in cortical neurogenesis (Liu et al., 2015). This suggests that overexpression of *OLIG2* in DS may (i) reduce the acquisition of a neuronal phenotype by reducing the expression of proneural genes and (ii) concurrently impair proliferation by reducing the expression of NFAT (Figure 7A). Experiments in cultures of DS-derived NPCs revealed an OLIG2-dependent reduction in the expression of KCNA3 potassium channel, suggesting that a decline in K^+^ channel activity may cause an elongation of the cell cycle and, thus, diminish NPC proliferation (Lu et al., 2012; Figure 7A). Intriguingly, during embryonic development, interneural precursors in the medial ganglionic eminence of the Ts65Dn mouse exhibit a faster proliferation rate, which is at variance with other neurogenic niches, although they exhibit higher expression levels of OLIG2 (and OLIG1), and this defect is abrogated by deletion of an allele of *Olig1* and *Olig2* (Chakrabarti et al., 2010). This suggests that OLIG1 and OLIG2 may play a differential role in the modulation of neurogenesis according to brain region and developmental time. A faster proliferation rate of interneuron precursors might translate into the increase in the number of calretinin-positive interneurons, a population that appears early in cortical development (see Bayatti et al., 2008), observed in fetuses (Guidi et al., 2018), and infants (Xu et al., 2019) with DS, in the Ts65Dn model (Perez-Cremades et al., 2010; Hernandez-Gonzalez et al., 2015) and in an alcohol syndrome model (Granato, 2006).

#### *IFNAR1, IFNAR2, IFNGR2*, and *IL10RB*

*IFNAR1*, *IFNAR2*, *IFNGR2*, which encode receptors for interferons (IF), and *IL10RB* which encodes a receptor for interleukin (IL) form a cluster on HSA21. Binding of IFNs and IL to their receptors activates JAK proteins that phosphorylate the transcription factors STATs that can migrate to the nucleus. Among the STATs, STAT3 specifies glial cell fate through transcriptional activation of astrocytic genes, such as *GFAP* and *S100beta*. *IFNAR1*, *IFNAR2*, *IFNGR2*, and *IL10RB* are upregulated in DS fibroblasts, rendering them more sensitive to interferon and inducing greater activation of the IFN pathways (Sullivan et al., 2016). In addition, an increase in IFNAR2 proteins has been detected in the cerebral cortex of DS fetuses at GW19–21 (Ferrando-Miguel et al., 2003) and serum levels of IL-6 are increased in DS children (Corsi et al., 2006). Taken together, these data suggest that overstimulation of JAK-STAT signaling due to overexpression of IFNRs and IL10R may promote NPC fate toward astrogliogenic pathways in DS (Figure 7B). Trisomic fibroblasts show activation of IFN ligands including IFNG (Sullivan et al., 2016), which reduces differentiation of oligodendrocyte precursors into oligodendrocytes and favors acquisition of an astrocytic phenotype (Tanner et al., 2011). This suggests that the JAK-STAT pathway may also increase astrogliogenesis at the expense of oligodendrocyte differentiation.

#### Amyloid Beta Precursor Protein

Repression of the SHH pathway due to APP-AICD mediated *PTCH1* overexpression (see above) causes downregulation of GLI transcription factors. GLI2 induces neurogenesis in neuronal stem cells by positively regulating the expression of neurogenic genes, such as *MASH1* (Voronova et al., 2011), suggesting that APP-AICD-mediated downregulation of GLI2 may reduce neurogenesis by reducing the expression of *MASH1* (Figure 7C). AICD can promote the expression of miR-665 in the nucleus which suppresses neuronal differentiation by reducing the expression of proneurogenic genes such as *FBXL18* and *CDK6* (Shu et al., 2015). This provides an additional mechanism whereby AICD may reduce neurogenesis (Figure 7C). Interaction between APP and S100beta promotes a deleterious pathway that causes oxidative stress (Lu et al., 2011); reactive oxygen species activate JNK/p38 and the JAK/STAT signaling pathway (Esposito et al., 2008). In addition, sAPP enhances the activity of the JAK-STAT signaling cascade (Trazzi et al., 2013). This evidence suggests that APP, in addition to reducing neurogenesis, favors astrogliogenesis (Figure 7B).

#### Dual Specificity Tyrosine-Phosphorylation-Regulated Kinase 1A

Overexpression of *Dyrk1a* in wild-type cortical progenitors increases STAT3 phosphorylation at Ser(727), which enhances the transcriptional activity of STAT3 (Kurabayashi et al., 2015), suggesting that increased dosage of *DYRK1A* may contribute to the gliogenic shift in DS (Figure 7B). Indeed, targeting DYRK1A pharmacologically or by shRNA in DS-hiPSCs resulted in a considerable correction in the acquisition of a neuronal phenotype (Hibaoui et al., 2014).

#### Early Undifferentiated Retina and Lens (*C21/ORF91*)

The gene Early Undifferentiated Retina and Lens (*EURL*), also called Chromosome 21 open reading frame 91 (*C21ORF91*) is a protein coding gene localized on Chr21 that is emerging as a potential candidate for neurogenesis impairment in DS. *EURL* is expressed in the fetal brain (GW16) and its transcripts undergo a temporal increase in neonatal and adult brains with a spatiotemporal profile that differs between DS and controls (Li et al., 2016). In a mouse model, knockdown of *Eurl* causes a reduction in radial glial progenitors (PAX6-positive cells) but not in NPCs (TBR2-positive cells; Li et al., 2016). Contrariwise, forced *Eurl* expression increases both progenitor populations. Moreover, both knockdown and enhancement of *Eurl* alter the cortical positioning of embryonically born neurons, indicating that the dose of *Eurl* is crucial for cortical development and neuron maturation. The significance of *EURL* overexpression in neurogenesis and neuron maturation in DS requires further investigation. A recent study examined the role of EURL in gliogenesis (Reiche et al., 2021). Results showed that forced overexpression of Eurl in cultured rat primary oligodendroglial precursor cells resulted in aberrant coexpression of astroglial and oligodendroglial markers. In particular, there was a reduction in the number of cells exhibiting oligodendroglial features, such as nuclear expression of OLIG2 and SOX10 and an increase in the number of cells exhibiting astrocytic features, such as ubiquitous (nuclear and cytoplasmatic) expression of OLIG2, which indicates astrogliogenesis (Setoguchi and Kondo, 2004) and Sox10, and increased expression of hairy and enhancer of split-1 (HES1) and GFAP (Reiche et al., 2021). This evidence suggests that *EURL* overexpression in DS may induce glial precursor cells to acquire an astrocytic phenotype at the expense of an oligodendroglial phenotype (Figure 7D). This conclusion is in line with the temporal profile of *EURL* expression, that peaks between birth and adulthood (Li et al., 2016), i.e., a time of prominent gliogenesis. Significantly, forced *Eurl* expression causes accelerated maturation of rat oligodendroglial cells but diminished myelination capacity (Reiche et al., 2021), suggesting that *EURL* plays a role in myelination impairment in DS.

## Genes Responsible for Neuronal Maturation Impairment in Down Syndrome

Evidence regarding this issue is currently very scarce, indicating the need for specific studies that focus on therapeutic interventions in the neonatal period, a critical window for neuronal maturation.

### Down Syndrome Cell Adhesion Molecule

Down syndrome cell adhesion molecule (*DSCAM*) is a gene located on the so-called critical region of Chr21, a region that was previously thought to be particularly relevant for the DS-linked phenotypes. *DSCAM* expression (RNA) is increased in neurospheres from GW8–GW18 fetuses (Bahn et al., 2002), and children and adults with DS exhibit higher brain DSCAM levels (Saito et al., 2000) compared to controls. During dendritic development DSCAM promotes self-avoidance through homophilic contact-mediated repulsion (Fuerst et al., 2008; Montesinos, 2017). Knockdown of DSCAM increases the complexity of dendritic branching and inhibits axon growth in mouse cortical neurons (Zhang et al., 2015). A recent study used a trisomic cell line (trisomic CTb, derived from Ts16 mice) to investigate the molecular mechanisms whereby DSCAM impairs development of neuritic processes (Perez-Nunez et al., 2016). This study shows that overexpressed DSCAM deregulates p21-activated kinase activity which, in turn, destabilizes actin cytoskeleton and formation of neuritic processes. DSCAM may also regulate neuron morphogenesis through its intracellular domain (ICD; Sachse et al., 2019). Gain-of-function experiments in primary cortical neurons show that increasing the levels of DSCAM or DSCAM ICD leads to an impairment of neurite growth and synapse number (Sachse et al., 2019).

### Regulator of Calcineurin 1

Human *RCAN1* transgenic mice, in which overexpression of RCAN1 was close to the level of overexpression observed in DS, exhibited reduced spine density on basal and apical dendrites of CA1 pyramidal neurons (Martin et al., 2012), suggesting that RCAN1 overexpression in DS may be involved in spinogenesis impairment.

### Early Undifferentiated Retina and Lens*//C21orf91*

Excessive levels of this gene cause various effects (see above), including reduction of dendritic spine density in a mouse model in which *Eurl* expression was enhanced (Li et al., 2016). The defects in spinogenesis in DS take place in infancy, during which EURL undergoes an increase in DS brains (Li et al., 2016), suggesting that this gene may concur to reduce dendritic spine density in DS children.

## Achievements Obtained by Early Pharmacotherapies in Down Syndrome Models

**FIGURE 8:**
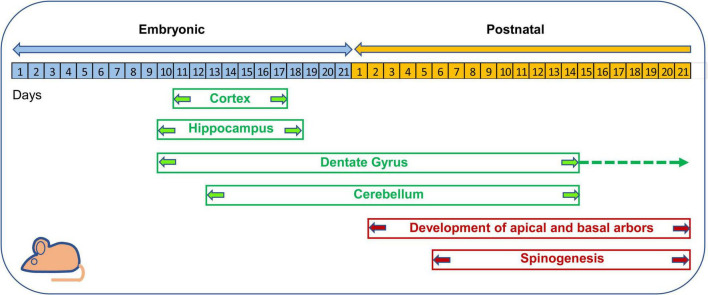
Timeline of neurogenesis and neuron maturation in the mouse brain. Cortical neurogenesis takes place between embryonic (E) days E11–E17 (Takahashi et al., 1996). In the hippocampus proper neurogenesis takes place between E10–E18 (Angevine, 1965). In the hippocampal DG, neurogenesis begins at E10, exhibits its maximum rate in the first two postnatal weeks and then continues at a slow rate throughout life (Altman and Bayer, 1975, 1990a,b). In the cerebellum, granule cell production begins at E12.5 and E15.5 and is accomplished by the second postnatal week (Sillitoe and Joyner, 2007; Sudarov and Joyner, 2007). The basal and apical arbors of cortical pyramidal neurons appear at postnatal day 2 and attain maturation within the third week (Meller et al., 1969; Uguagliati et al., 2022). Cortical spinogenesis begins at postnatal days 6–9 and is completed by the third postnatal week (Meller et al., 1969; Uguagliati et al., 2022).

During the past 20 years various studies have exploited mouse models of DS to establish whether it is possible to pharmacologically improve the morpho-functional brain defects of DS and behavior. Most of these studies have been carried out at adult life stages (see Costa and Scott-McKean, 2013; Gardiner, 2015; Stagni et al., 2015a; Hart et al., 2017; Vacca et al., 2019; Rueda et al., 2020a). This timing, however, is not suitable to counteract neurogenesis alterations, because in mice, similarly to humans, neurogenesis is a prenatal/neonatal event. Cortical neurogenesis occurs between E11–E17, hippocampal neurogenesis occurs between E10–E18, cerebellar granule cell neurogenesis starts at E13 and is completed at postnatal day 14 (Figure 8). Unlike the rest of the brain, the DG produces most of its neurons (∼80%) in the first two neonatal weeks and continues, slowly, to produce neurons throughout life (Figure 8). Dendritogenesis and spinogenesis occur from birth to weaning (Figure 8). Thus, considering the milestones of mice brain development, we report here only studies in which mice were treated during the embryonic (Table 1; 21 studies) and neonatal (Table 2; 19 studies) period. Tables 1, 2 summarize the type and timing of treatments, the short- and long-term effects of treatment on neuroanatomy and behavior, and, when available, the effects on molecular pathways. For ease of reference, treatments are labeled with a “T” followed by a number and individual studies are labeled with an “S” followed by a number. The substances used in the studies reported in Tables 1, 2 were either of non-natural or natural origin and were chosen based on a rationale detailed in the corresponding articles, to which the reader is referred. We will comment on these studies below, with the principal aim of highlighting the aspects that could serve as a guide for the design of fetal therapies for DS.

**TABLE 1:** Treatments administered at embryonic life stages in DS mouse models.

S	T	Treatment	Action	Treatment window	Age at testing	Effects	References
S1	T1	Fluoxetine	Inhibitor of serotonin reuptake	E10-Birth	Short term: P2 Long term: P45	NPC Proliferation (DG, SVZ, CX, STR, TH, HYP, MES, CRB): **R** Cellularity (CX, DG, CA3, CA1, STR, TH, HYP, MES, CRB): **R** NPC Proliferation (DG, SVZ): **R** Neurogenesis (DG): **R** Cellularity (DG, CX)**: R** Dendritic hypotrophy (DG): **R** Connectivity (CX, CA1, DG): **R** Fiber tract size: **R** p21, pERK1/2: **R** L/M (Contextual Fear Conditioning): **R**	Guidi et al., 2014
S2	T2	ALGERNON	Inhibitor of DYRK1A	**§** E10–E15	Short term: E15.5 Long term: Adult	Intermediate zone and cortical plate thickness: **R** NPC proliferation (DG): **F** L/M (Y Maze): **PR** L/M: (Barnes Maze): **PR** L/M (Contextual Fear Conditioning): **R**	Nakano-Kobayashi et al., 2017
S3	T3	Rapamycin	Inhibitor of mTOR	**§** E15 1 injection	Long term: P18 Long term: P21–P30	Spine density (CA1): **F** Mushroom spines: **R** LTD (CA1): **R**	Urbano-Gamez et al., 2021
S4	T4	SGS-111	Nootropic agent	E1–5M	Short term: 3M	Sensorimotor tests: F L/M (Morris Water Maze, Passive Avoidance): **F**	Rueda et al., 2008
S5	T5	NAP + SAL	Small peptides mimetic of ADNP and ADNF	E8–E13	Short term: P5–P21	Motor and sensory milestones: **R**	Toso et al., 2008
S6	T5	NAP + SAL	Small peptides mimetic of ADNP and ADNF	E8–E12	Long term: 8–10M	L/M (Morris Water Maze): **R**	Incerti et al., 2012
S7	T6	P021	Small-peptide mimetic of the ciliary neurotrophic factor	E1–P21	Short term: P1–P21 Long term: 5–7M	Motor and sensory milestones: **PR** pGSK3ß, pCREB, BDNF: **R** L/M (Novel Object Recognition): **PR** L/M (Morris Water Maze): **R** pGSK3ß, pCREB, BDNF: **R**	Kazim et al., 2017
S8	T7	Choline	Precursor of acetylcholine	E1–P21	Long term: 6–12M	Visual attention tasks: **PR**	Moon et al., 2010
S9	T7	Choline	Precursor of acetylcholine	E1–P21	Long term: 13–17M	Neurogenesis (DG): **PR** L/M (Radial Arm Water Maze): **PR**	Velazquez et al., 2013
S10	T7	Choline	Precursor of acetylcholine	E1–P21	Long term: 13–17M	ChAT-positive cells (septum): **PR** L/M (Radial Arm Water Maze): **PR**	Ash et al., 2014
S11	T7	Choline	Precursor of acetylcholine	E1–P21	Long term: 4–7M	Number of basal forebrain cholinergic neurons: **PR** Cholinergic innervation (hippocampus): **PR**	Kelley et al., 2014
S12	T7	Choline	Precursor of acetylcholine	E1–P21	Long term: 12M Long term: 16M	Attention tasks: **PR** Attention tasks: **F**	Powers et al., 2021
S13	T8	Melatonin	Hormone	E1–4.5/5M	Short term: 4.5–5M	NPC proliferation (DG): **F** Cellularity (DG): **F** Sensorimotor tests: **F** L/M (Contextual Fear Conditioning, Morris Water Maze): **F**	Corrales et al., 2017
S14	T9	α-tochopherol	Antioxidant	E1–12W	Short term: 12W	Cellularity (DG): **R** Anxiety (Elevated-plus maze): **PR** L/M (Morris Water Maze): **PR**	Shichiri et al., 2011
S15	T10	EGCG enriched green tea extract	DYRK1A natural inhibitor and antioxidant	E1–5M	Short term: 5M	L/M (Morris Water Maze): **PR**	Yin et al., 2017
S16	T10	EGCG enriched green tea extract	DYRK1A natural inhibitor and antioxidant	**§§** E1–P90	Short term: P90	L/M (Y Maze): **F** L/M (Novel Object Recognition*):* **PR**	Souchet et al., 2019
S17	T11	Apigenin 4,5,7-trihydroxyflavone	Antioxidant	**§** E1–P21	Short term: E15.5 Short term: P3–P21 Long term: Adults	Overexpression of *Dscam*, *Kcnj6*, *Pcp4*, *Ets2*, *Il10rb*, *Cav1*, *Dtna*: **PR** Upregulation of proneural genes (e.g., *Nestin*, *Sox2*, *Pax6)* Developmental milestones*:* **PR** Olfactory memory*:* **PR** L/M (Contextual Fear Conditioning*):* **R**	Guedj et al., 2020
S18	T12	7,8-dihydroxyflavone	BDNF mimetic and antioxidant	E10-Birth	Short term: P2 Long term: P52–P60	NPC proliferation (DG, SVZ, CX, STR): **R** NPC proliferation (TH, HYP): **F** Cellularity (CX): **R** Cellularity (DG, CA1): **F** NPC proliferation (DG): **F** Neurogenesis (DG): **R** Cellularity (DG): **PR**	Stagni et al., 2021
S19	T13	Curcumin	Pleiotropic effects	E10–P2	Short term: P2 Long term: P45	NPC proliferation (DG): **R** Cellularity (DG): **F** Connectivity (DG, CA1, CA3): **PR** NPC proliferation (DG): **F** Cellularity (DG): **F** Connectivity (DG, CA1, CA3): **F** L/M (Morris Water Maze): **PR**	Rueda et al., 2020b
S20	T14	Oleic acid	Monounsaturated fatty acid of the Ω9 series that occurs naturally in fats	E10–P2	Short term: P2 Long term: P45	NPC proliferation (DG): **R** Cellularity (DG): **R** NPC proliferation (DG): **F** Connectivity (DG, CA1, CA3): **R** L/M (Morris Water Maze): **R**	Garcia-Cerro et al., 2020
S21	T15	Linolenic acid	Polyunsaturated fatty acid of the Ω3 series that occurs naturally in fats	E10–P2	Short term: P2 Long term: P45	NPC proliferation (DG): **R** Cellularity (DG): **R** NPC proliferation (DG): **F** Connectivity (DG, CA1, CA3): **PR** L/M (Morris Water Maze): **R**	Garcia-Cerro et al., 2020

*Summary of the main effects of embryonic treatment in Ts65Dn, Ts1Cje (labeled with § in the column “Treatment Window”), and Dp(16) (labeled with §§ in the column “Treatment Window”) mice. The 15 substances used for treatment (T) tested in prenatal studies (S1–S21) have been grouped as follows: T1–T6 are non-natural substances and T7–T15 are natural substances. The non-natural substances T5 and T6 are peptides of neurotrophic factors: NAP and SAL are fragments of the activity dependent neuroprotective protein (ADNP) and activity dependent neurotrophic factor (ADNF), respectively; P021 is a peptide of the ciliary neurotrophic factor. The reported studies examined the short-term and/or long-term effects of treatment at the ages indicated in the columns “Age at Testing”. One or more of the following variables were investigated: NPC proliferation, neurogenesis, cellularity, connectivity (i.e., density of pre- and postsynaptic terminals), dendritic arborization, spine density, long-term depression, and behavior. A few studies also examined molecular mechanisms. The effects of treatment are indicated as follows; R, Rescue; PR, Partial Rescue; and F, Failure. Abbreviations: ChAT, Choline acetyltransferase; CRB, cerebellum; CX, cortex; DG, dentate gyrus; E, embryonic; HYP, hypothalamus; L/M, learning and memory; LTD, long-term depression; M, month; MES, mesencephalon; NPC, neural progenitor cells; P, postnatal; STR, striatum; S, study; SVZ, subventricular one; T, treatment; and TH, thalamus.*

**TABLE 2:** Treatments administered at neonatal life stages in the Ts65Dn model.

S	T	Treatment	Action	Treatment window	Age at testing	Effects	References
S1	T1	SAG	Sonic Hedgehog pathway agonist	P0 1 Injection	Short term: P6	NPC proliferation (Cerebellum): **R** Cellularity (Cerebellum): **R**	Roper et al., 2006
S2	T1	SAG	Sonic Hedgehog pathway agonist	P0 1 Injection	Short term: P6 Long term: 4M	NPC proliferation (DG): **F** Cellularity (Cerebellum): **R** Cerebellar size: **R** LTD (Cerebellum): **F** LTP (CA1): **R** L/M (Y Maze): **F** L/M (Morris Water Maze): **R**	Das et al., 2013
S3	T1	SAG	Sonic Hedgehog pathway agonist	P0 1 Injection	Long term: 4M	Cerebellar size: **R** Cellularity (Cerebellum): **R** Cerebellar functional deficits: **F**	Gutierrez-Castellanos et al., 2013
S4	T2	Fluoxetine	Inhibitor of serotonin reuptake	P3–P15	Short term: P15 Long term: P45	NPC proliferation (DG, SVZ): **R** Cellularity (DG): **R** BDNF: **R** NPC proliferation (DG): **R** Neurogenesis (DG) **R** Cellularity (DG): **R** L/M (Contextual Fear Conditioning): **R**	Bianchi et al., 2010
S5	T2	Fluoxetine	Inhibitor of serotonin reuptake	P3–P15	Long term: P45	Dendritic hypotrophy (DG): **R** Spine density (DG): **R** Connectivity (DG): **R** DSCAM: **R**	Guidi et al., 2013
S6	T2	Fluoxetine	Inhibitor of serotonin reuptake	P3–P15	Long term: P45	Dendritic spines (CA3): **R** DG- > CA3 input: **R** EPSCs and IPSCs (CA3): **R**	Stagni et al., 2013
S7	T2	Fluoxetine	Inhibitor of serotonin reuptake	P3–P15	Long term: 2.5M	NPC proliferation (DG, SVZ): **R** Cellularity (DG): **R** Neurogenesis (DG); **R** Dendritic hypotrophy (DG): **R** Spine density (DG): **R** Connectivity (DG): **R** L/M (Morris Water Maze, Novel Object Recognition, Passive Avoidance): **R** p21, BDNF, ERK1/2, ß-secretase, ßCTF: **R**	Stagni et al., 2015b
S8	T3	ELND006	γ-secretase inhibitor	P3–P15	Short term: P15	NPC proliferation (DG, SVZ): **R** Cellularity (DG): **R** Connectivity (DG, CA3): **R** APP, PTCH1; pGSK3ß: **R**	Giacomini et al., 2015
S9	T3	ELND006	γ-secretase inhibitor	P3–P15	Long term: P45	NPC proliferation (DG): **R** Cellularity (DG): **R** Connectivity (DG): **F** Connectivity (CA3): **R** EPSCs (CA3): **R** p21: **PR**	Stagni et al., 2017b
S10	T4	Cyclosporin A	Calcineurin inhibitor	P3–P15	Short term: P15	NPC proliferation (DG, SVZ): **R** Cellularity (DG): **R** Spine density (DG): **R** p21: **R**	Stagni et al., 2019b
S11	T5	Clenbuterol	ß2 adrenergic receptor agonist	P3–P15	Short term: P15	NPC proliferation (DG): **R** Cellularity (DG): **PR** Dendritic hypotrophy: **R** Spine density (DG): **R**	Emili et al., 2020
S12	T6	Salmeterol	ß2 adrenergic receptor agonist	P3–P15	Short term: P15	NPC proliferation (DG): **F** Dendritic hypotrophy: **R** Spine density (DG): **R**	Emili et al., 2020
S13	T7	EGCG	DYRK1A natural inhibitor and antioxidant	P3–P15	Short term: P15 Long term: P45	NPC proliferation (DG, SVZ): **R** Cellularity (DG): **R** Connectivity (DG, CA1; CX): **R** p21, pGSK3ß: **R** NPC proliferation (DG): **F** Neurogenesis (DG): **F** Cellularity (DG): **F** Connectivity (DG, CA1; CX): **F** pGSK3ß: **F** L/M (Morris Water Maze): **F**	Stagni et al., 2016
S14	T8	7,8-dihydroxyflavone	BDNF mimetic and antioxidant	P3–P15 P3–P45	Short term: P15 Short term: P45	NPC proliferation (DG): **PR** Cellularity (DG): **R** Spine density (DG): **R** BDNF: **F** pERK1/2: **R** L/M (Morris Water Maze): **R**	Stagni et al., 2017a
S15	T8	7,8-dihydroxyflavone	BDNF mimetic and antioxidant	P3–P15	Long term: P45	Neurogenesis (DG): **F** L/M (Morris Water Maze): **F**	Giacomini et al., 2019
S16	T8	7,8-dihydroxyflavone	BDNF mimetic and antioxidant	P3–P15	Short term: P15	Mitochondrial function: **R** PGC-1α: **R**	Valenti et al., 2021
S17	T9	Curcumin	Pleiotropic effects	P3–P15	Short term: P15 Long term: 3M	NPC proliferation (DG): **F** Cellularity (DG): **F** Connectivity (CA3): **PR** NPC proliferation (DG): **F** Cellularity (DG): **F** Connectivity (CA3): **F** L/M (Morris Water Maze): **F**	Rueda et al., 2020b
S18	T10	Oleic acid	Monounsaturated fatty acid of the Ω9 series that occurs naturally in fats	P3–P15	Short term: P15 Long term: 3M	NPC proliferation (DG): **F** Cellularity (DG): **R** Connectivity (DG, CA1, CA3): **R** NPC proliferation (DG): **F** Neurogenesis (DG): **R** Cellularity (DG): **F** Connectivity (DG, CA1, CA3): **PR** L/M (Morris Water Maze): **R**	Vidal et al., 2020
S19	T11	Linolenic acid	Polyunsaturated fatty acid of the Ω3 series that occurs naturally in fats	P3–P15	Short term: P15 Long term: 3M	NPC proliferation (DG): **F** Cellularity (DG): **F** Connectivity (DG, CA1, CA3): **PR** NPC proliferation (DG): **F** Neurogenesis (DG): **F** Cellularity (DG): **F** Connectivity (DG, CA1, CA3): **PR** L/M (Morris Water Maze): **PR**	Vidal et al., 2020

*Summary of the main effects of neonatal treatment in Ts65Dn mice. There are no similar studies for other DS models. The 11 substances used for treatment (T) tested in neonatal studies (S1–S19) have been grouped as follows: T1–T6 are non-natural substances and T7–T11 are natural substances. The non-natural substances T4, T5, and T6 are drugs: cyclosporine (T4) is used as an immunosuppressant and clenbuterol (T5) and salmeterol (T6) are used for the treatment of asthma. The reported studies examined the short-term and/or long-term effects of treatment at the ages indicated in the columns “Age at Testing.” P0 corresponds to the day of birth. One or more of the following variables were investigated: NPC proliferation, neurogenesis, cellularity, connectivity (i.e., density of pre- and postsynaptic terminals), dendritic arborization, spine density, long-term potentiation, long-term depression, and behavior. A few studies also examined molecular mechanisms. The effects of treatment are indicated as follows; R, Rescue; PR, Partial Rescue; and F, Failure. Abbreviations: CX, cortex; DG, dentate gyrus; EGCG, epigallocatechin-3-gallate; EPSCs, excitatory postsynaptic currents; IPSCs, inhibitory postsynaptic current; L/M, learning and memory; LTD, long-term depression; LTP, long-term potentiation; M, month; NPC, neural progenitor cells; P, postnatal; and SVZ, subventricular zone.*

### Prenatal Studies

#### Timing

Non-invasive prenatal testing (NIPT), based on analysis of cell free DNA circulating in the maternal plasma, allows trisomy of the fetus to be established with good confidence (Mersy et al., 2013). This test takes place no earlier than 10–11 weeks into pregnancy. Treatments in mice that started at conception (Table 1: S4, S7–S17) are logical, because they cover the whole period of neurogenesis, but this strategy does not mimic what would happen in the case of trisomy 21 diagnosis, which is necessarily delayed. From this viewpoint, studies that started at later times of gestation (Table 1: all other studies) may provide better insight for human application.

#### Type of Treatment and Short- and Long-Term Effect on Neural Progenitor Cell Proliferation/Neurogenesis

Only 8 out of 21 studies examined the effect of treatment on NPC proliferation and/or neurogenesis (S1, S2, S9, S13, S18–S21). These studies used either natural or non-natural substances. The type of treatment represents a “hot” issue considering potential side effects, especially during pregnancy. From this viewpoint, natural substances, which, at proper doses have a safe profile, may be preferable. A comparison of the studies that examined NPC proliferation/neurogenesis shows that natural substances (save for melatonin, that has no effect on any examined variable; Table 1: S13) have a short-term positive effect on proliferation/neurogenesis (Table 1: S9, S18–S21) similarly to fluoxetine, the only non-natural substance for which short-term effects are available (Table 1: S1). While the effect of fluoxetine was retained in adulthood (Table 1: S1), the effects of natural substances on proliferation/neurogenesis disappeared with time (Table 1: S18–S21), with the exception of 7,8-dihydroxyflavone (7,8-DHF; Table 1: S18).

#### Treatment Effects Beyond Neurogenesis

Some of the prenatal studies examined one or more of the following variables: cellularity, dendritic hypotrophy, spine density, and connectivity. Both natural and non-natural substances were effective. However, the long-term effects of natural substances (Table 1: S18–S21), unlike those of non-natural substances (Table 1: S1, S3), diminished or disappeared with time. Regarding the effect on behavior, save for two studies (S4 and S13), studies that examined learning and memory (L/M) report rescue or a partial rescue (Table 1: S1, S2, S6, S7, S9, S10, S14–S17, S19–S21). Interestingly, although the long-term beneficial effects of natural substances on neurogenesis fade with time, L/M is restored or improved in adulthood (Table 1: S19–S21).

### Neonatal Studies

#### Timing

Treatments reported in Table 2 covered the first two postnatal weeks, i.e., the period of maximum hippocampal neurogenesis in rodents. In mice, the dendritic spurt and appearance of dendritic spines takes place in the first two postnatal weeks (Figure 8). The first two postnatal weeks in mice, therefore, correspond with the third trimester of gestation in humans (Figure 2). This correspondence also holds for other neurodevelopmental aspects (Clancy et al., 2001). Thus, from a translational viewpoint, the effect of neonatal treatment in mice may partially mimic treatments during late gestation in humans.

#### Type of Treatment and Short- and Long-Term Effect on Neural Progenitor Cell Proliferation/Neurogenesis

Six out of the 11 molecules reported in Table 2 are of non-natural origin (T1–T3) or are drugs (T4–T6), and 5 are natural substances (T7–T11). Fifteen out of the reported studies (19) examined hippocampal NPC proliferation and/or neurogenesis (S1, S2, S4, S7–S15, and S17–S19). A comparison of these studies shows that 5 out of 6 non-natural substances (T1–T5) and 2 out of six natural substances (T7 ad T8) rescued hippocampal proliferation/neurogenesis. Save for oleic acid (S18), natural substances did not have a long-term effect (S13, S15, S17, and S19). In contrast, the 3 studies that examined long-term effects with non-natural substances found a long-term benefit on NPC proliferation/neurogenesis (S4, S7, and S9).

#### Treatment Effects Beyond Neurogenesis

Many of the studies in Table 2 examined cellularity, dendritic hypotrophy, spine density, and connectivity. Both non-natural (S1–S12) and natural (S13, S14, and S17–S19) substances rescue or partially rescue these defects. However, while non-natural substances exert long-term effects (S2–S7, S9), the effects of natural substances are not retained (S13, S15, and S17) or are attenuated (S18, S19). Regarding learning and memory (L/M), both non-natural (S2, S4, and S7) and natural (S14, S18, and S19) substances may exert a positive effect, although some natural substances do not elicit any behavioral improvement (S13, S17).

### Lesson Learned From Early Treatments in Mouse Models

The studies in DS models reported in Tables 1, 2 unequivocally show that prenatal or postnatal treatment can restore neurogenesis, cellularity, connectivity, dendritogenesis, and behavior, indicating that the major DS-linked brain defects can be pharmacologically improved. Treatment with both natural and non-natural substances was effective, although the benefit of natural substances tended to fade away with time, especially in the case of postnatal treatment. Some treatments (fluoxetine, 7,8-DHF, curcumin, oleic acid, and linolenic acid) were tested in the prenatal (Table 1: S1, S18–S21) and neonatal (Table 2: S4–S7, S14–S19) period, allowing comparison of the same therapy during different time windows. (i) Fluoxetine emerges as the only treatment exerting equally powerful effects both prenatally and postnatally; (ii) Prenatal treatment with 7,8-DHF, curcumin, oleic acid, and linolenic acid leaves a larger trace in the brain in comparison with postnatal treatment; and (iii) oleic acid (Table 1: S20) and 7,8-DHF (Table 1: S18) result more effective than curcumin and linolenic acid. The larger efficacy of embryonic treatment is not unexpected, considering that trisomy-linked brain defects start during prenatal life stages. Thus, a fetal therapy (with the right treatment) may be much more beneficial for DS than postnatal therapy.

Considering the heterogeneity of treatment employed in mouse models, it is somewhat surprising that such a variety of agents has a similar outcome. Tables 1, 2 show that both natural (Table 1: S17 and Table 2: S13, S14) and non-natural (Table 1: S1, S7 and Table 2: S7–S10) substances restored molecular mechanisms involved in neurogenesis alterations in DS (e.g., p21 levels or GSK3ß phosphorylation), indicating, significantly, that it is possible to bypass pharmacologically triplicated genes and that this action may be achieved with a variety of agents.

An obvious question regard which of the treatments attempted in mice may now be reasonably proposed for humans (this issue is also discussed below). Since DYRK1A appears to be a key determinant of neurogenesis alterations, it may represent a suitable therapeutic target. Embryonic treatment with a DYRK1A inhibitor (Table 1: S2) restored the thickness of neurogenic niches and restored/improved L/M, although it did not restore DG neurogenesis in the Ts1Cje model. This treatment, however, restored proliferation of NPCs derived from individuals with DS (Nakano-Kobayashi et al., 2017). Postnatal treatment with EGCG, which is a DYRK1A inhibitor (and antioxidant) restores NPC proliferation and connectivity, but its effects are completely extinguished 1 month later (Table 2: S13). This suggests the necessity to screen additional DYRK1A inhibitors in preclinical studies. Among the non-natural substances, fluoxetine resulted the most potent in terms of scope and duration of its effects. Its use in pregnancy, however, may raise concern due to potential side effects (discussed in Stagni et al., 2015a), although, in view of its pediatric use, it may be proposed as treatment during postnatal time windows. Importantly, various natural substances proved effective (in particular oleic acid and 7,8-DHF) which, in view of their safe profile, makes them ideal candidates for prenatal treatment. Whatever the choice, the preclinical evidence provides strong support to the idea that treatments for the improvement of neurogenesis (and other defects) in DS are feasible.

## Long-Term Perspectives for Treatment

Neither prenatal nor postnatal treatments are currently available. However, thanks to the enormous progress in deciphering the molecular mechanisms of neurogenesis alterations in DS and knowledge that neurogenesis can be pharmacologically improved in DS models, the path leading to fetal (and neonatal) therapy is beginning to be better delineated and perspectives for treatment are becoming progressively more realistic. Which are the steps that should guide future actions?

### The Scenario: The Timing of Treatment Is Well-Delineated

**FIGURE 9:**
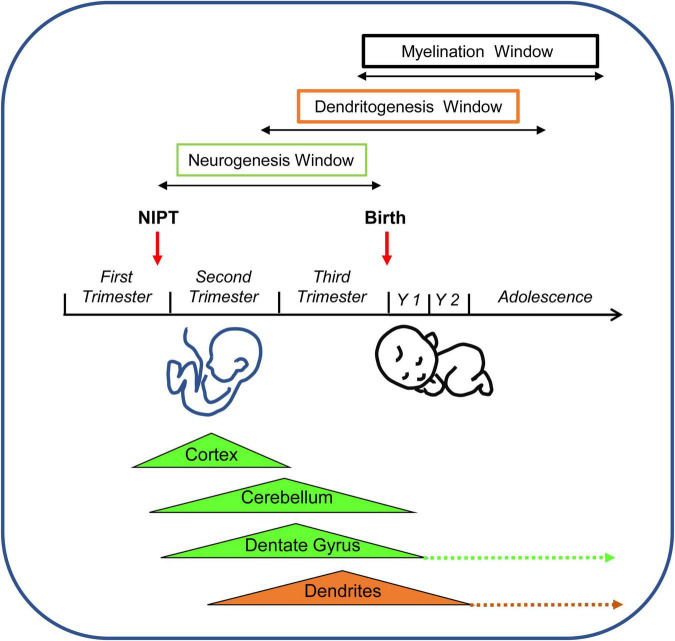
Windows of opportunity for the improvement of neurogenesis, dendritogenesis, and myelination in DS. Note that the prenatal and postnatal timelines are not to scale. The time at which NIPT is generally performed is indicated. Abbreviations; NIPT, non-invasive prenatal testing; Y, year.

It is now clear that in DS fetuses neurogenesis defects are already present at GW17 and very likely begin earlier. Defects in dendritogenesis appear in infancy and defects in myelination begin prenatally but become more prominent in adolescence. This knowledge provides windows of opportunity within which to counteract each of these defects. Interventions targeting cortical neurogenesis should be performed before the end of the second trimester. Interventions during late gestation might change cerebellar and hippocampal neurogenesis only. Interventions in adulthood might also modify hippocampal neurogenesis, although the small size of the postnatally proliferating population cannot radically change hippocampal cellularity. Treatment during late gestation and infancy/adolescence may be used to improve dendritogenesis and myelination (Figure 9). This scenario provides a rational basis for the timing of specific interventions. Considering that deficits in neuron number are most likely a leading cause of ID in DS, prenatal treatment counteracting neurogenesis impairment are likely to have a very large impact on ID. Therefore, we will focus here on the problems posed by prenatal interventions only. The pressing questions now are: (1) what kind of treatment? (2) which steps could promptly lead from “bench to bedside”?

### The Big Challenge for the Future: What Kind of Therapy?

The studies in DS mouse models summarized above show that various treatments are effective in improving or restoring prenatal neurogenesis. The question now is: what is the rational and ethical basis for choosing a treatment to be administered to mothers who are expecting a baby with DS? There are two options: treatments targeting triplicated genes that are known to disrupt neurogenesis (targeted treatments) or “generic” treatments with molecules that foster neurogenesis in the normal (or diseased) brain (untargeted treatments).

#### Targeted Treatments: The Pros and Cons

Since different triplicated genes concur to impair neurogenesis, which is/are the best candidate/s? To answer this question, we need to (i) examine the scope of their effects; (ii) establish whether there are drugs that counteract their activity; and (iii) have a clear picture of their temporal expression profile in the fetal DS brain. Based on the extent of their effects, *DYRK1A* and *APP* result as the best candidates because their overexpression reduces proliferation and neurogenesis, and increases gliogenesis, through a variety of mechanisms. *RCAN1* also plays a role in neurogenesis impairment in DS, although less multifaceted. While there is no specific drug targeting RCAN1, drugs are available that target DYRK1A and APP. Regarding DYRK1A, numerous inhibitors of its kinase activity are at hand (Nguyen et al., 2017; Atas-Ozcan et al., 2021), and a natural inhibitor of DYRK1A, EGCG, has been used in a pilot study in young adults with DS (De la Torre et al., 2014). Regarding APP, its small derivative AICD is the material effector of neurogenesis disruption, suggesting that drugs preventing AICD formation may be a useful strategy. Indeed, treatment with a γ-secretase inhibitor, that reduces the cleavage of ß-CTF and, consequently, AICD production, fully restores hippocampal neurogenesis in Ts65Dn mice (Giacomini et al., 2015). However, the fact cannot be ignored that inhibition of ß-CTF cleavage results in its accumulation which may cause endosome abnormalities and foster development of AD (Kim et al., 2016). Luckily, recent evidence in adult Ts65Dn mice shows that it is possible to reduce the level of APP itself (and, thus, of its derivative) through administration of posiphen, a translational inhibitor of APP, with no adverse effects (Chen et al., 2021). Importantly, posiphen fosters hippocampal neurogenesis and stimulates dendritic arborization in a model of AD (Lilja et al., 2013), indicating that reduction of APP levels restores two typical defects of DS. Thus, the possibility of preventing APP synthesis also makes it worth considering APP-targeted therapy. An ongoing clinical trial is testing the safety of posiphen in subjects with early AD^1^. Regarding the expression profile of *DYRK1A* and *APP* in the fetal DS brain, relatively scarce and sparse data are available (see section “Genes Responsible for Neurogenesis Impairment in Down Syndrome”). Knowledge of the timeline of *DYRK1A* and *APP* overexpression is mandatory to time fetal treatment correctly. These gaps still remain. The importance of a clear picture of the temporal expression of candidate genes is exemplified by the contradictory results obtained in Ts65Dn mice following postnatal treatment with EGCG at different time points. While neonatal treatment restored hippocampal neurogenesis (Stagni et al., 2016), slightly later treatments were of no benefit (Stringer et al., 2015, 2017), very likely due to changes in DYRK1A expression. At this point there is an additional issue to be considered. Fetal therapies imply that treatment must be administered to the mother. Thus, the question arises: do treatments targeting a triplicated gene (such as DYRK1A or APP) pose safety concerns for the expectant mother who, unlike the fetus, has two copies of it? The possibility that reduction of DYRK1A activity or APP levels may destabilize the neurochemistry of the mother cannot be ignored. To overcome this hurdle, we need specific preclinical studies in mouse models devoted to thorough investigations on the immediate and long-term effects of treatment on the dam. The caveats outlined above pose hurdles that cannot be readily overcome and whose removal will require time and intense effort.

#### Untargeted Treatments: The Pros and Cons

Although treatments targeting triplicated genes are, theoretically, the best choice, their application for human use during pregnancy may require years. Treatment employing a different strategy may result in a more prompt, feasible, and safe application. Natural compounds inducing neurogenesis and neuronal differentiation are presently attracting extensive attention (see An et al., 2021). These include polyphenols, flavonoids, glucosides, etc. Natural compounds can regulate the expression of (i) proteins involved in NPC proliferation and neurogenesis, such as STAT3, HES1, NEUROD1, NOTCH, and cyclin D1, (ii) transcription factors such as NGN1, and (iii) signaling pathways such as JAK/STAT, GSK-3β/β-catenin, to mention only some of their effects. Natural compounds have been proven to restore neurogenesis (and other DS defects) in DS mouse models (see Tables 1, 2). Among these, apigenin which is a natural flavone, has been shown to ameliorate deranged signaling pathways, (e.g., STAT and interferon signaling) and to induce overexpression of genes associated with G2/M cell-cycle transition in trisomic amniocytes (Guedj et al., 2020). Moreover, in Ts1Cje mice prenatally treated with apigenin, the expression of genes that negatively affect neurogenesis, such as *Dscam*, is partially corrected and the expression of genes implicated in neural stem cell proliferation, such as *Nestin*, *Sox2*, and *Pax6*, and of proneural genes, such as *Neurog1* and *Neurog2*, is significantly upregulated (Guedj et al., 2020). Moreover, luteolin, the major metabolite of apigenin, promotes hippocampal neurogenesis and increases the expression of *Nestin* in adult Ts65Dn mice (Zhou et al., 2019). Neonatal treatment with the natural flavonoid 7,8-DHF activates Erk1/2 signaling (Stagni et al., 2021), which regulates cyclin D1 transcriptional induction (Chambard et al., 2007). Both apigenin and 7,8-DHF, as well as the other natural substances used in DS models (Tables 1, 2), have no known toxicities suggesting safe use in pregnancy. Although the effects of natural substances tend to disappear with time, in view of their safe profile protocols of periodic treatments may be envisaged for the maintenance of their effects. Albeit the effects of natural substances may be less powerful in comparison with targeted therapies, even a small improvement of ID might be considered a success. Therefore, we believe that the use of natural compounds for prenatal treatment in DS is an avenue that should be intensely explored because it may lead to a quicker transfer from preclinical evidence to clinical trials.

#### Cerebral Organoids: A Key Platform for Treatment Selection

Although studies in animal models represent a fundamental step for development of therapies for human diseases, adding the use of screens in human cells would provide a more robust starting platform, especially in the case of prenatal therapies. Cerebral organoids derived from hiPSCs represent the best possible approximation of a whole brain and may represent a powerful tool for studying the biology of DS (and other neurological disorders) and potential treatments (Tambalo and Lodato, 2020; Samarasinghe et al., 2021; Tang et al., 2021). The recent demonstration that it is possible to create hiPSC-derived organoids that closely reproduce dorsal forebrain identity^2^, suggests the possibility to exploit brain organoids to approximate prenatal and early postnatal development and test the efficacy and safety of drugs at these developmental stages. Such an approach may represent an invaluable tool for the selection of treatments for DS and the design of clinical trials during the delicate periods of pregnancy and in infancy.

### Ethical Considerations

The sensitivity and accuracy of NIPT consents timely identification of DS, thereby enabling expectant mothers to decide whether to interrupt pregnancy. They could be guided in this morally difficult decision by knowledge that treatment options exist that may mitigate the consequences of Chr21 triplication. This and other ethical issues have been examined in a recent publication to which the reader is referred (de Wert et al., 2017). We will limit ourselves here to the following consideration: let us take the case of a mother who decides not to have an abortion. Knowledge that a fetal drug therapy is feasible and awareness that omission of treatment may deprive her baby of the opportunity for a better life are likely to create a moral dilemma. Natural substances have been traditionally used by mankind for their beneficial effects in a variety of illnesses and absence of side effects (at proper doses). Given that different treatment options are available, the possibility to choose treatment with natural substances might greatly facilitate the decision regarding “treatment or no treatment” and direct the choice toward treatment, with possible benefits for the unborn child.

## Conclusion

The high prevalence of DS, the increase in longevity of people with DS and the associated risk of AD are increasing the burden of this disease on families and society. The already available and compelling demonstration that it is possible to rescue neurogenesis in mouse models of DS opens the possibility to achieve similar benefits in humans. Although the long-term perspectives for treatment delineated above contain various obstacles, we believe that these obstacles are not insurmountable provided that investment in terms of economical and human resources are undertaken. We hope that the large body of evidence showing that the neurodevelopmental defects of DS are amenable to treatment will draw the attention of institutions and stakeholders, thereby fostering the creation of a world-wide consortium devoted to the prevention of ID in DS.

## Author Contributions

RB: conceptualized the review. RB and FS wrote the review. Both authors contributed to the article and approved the submitted version.

## Conflict of Interest

The authors declare that the research was conducted in the absence of any commercial or financial relationships that could be construed as a potential conflict of interest.

## Publisher’s Note

All claims expressed in this article are solely those of the authors and do not necessarily represent those of their affiliated organizations, or those of the publisher, the editors and the reviewers. Any product that may be evaluated in this article, or claim that may be made by its manufacturer, is not guaranteed or endorsed by the publisher.

## Footnotes

1
https://clinicaltrials.gov/ct2/show/NCT02925650

2
https://www.biorxiv.org/content/10.1101/2022.02.28.482350v1

## References

[B1] Abraham H. Tornoczky T. Kosztolanyi G. Seress L. (2001). Cell formation in the cortical layers of the developing human cerebellum. *Int. J. Dev. Neurosci.* 19 53–62. 10.1016/s0736-5748(00)00065-4 11226755

[B2] Abraham H. Vincze A. Veszpremi B. Kravjak A. Gomori E. Kovacs G. G. (2011). Impaired myelination of the human hippocampal formation in Down syndrome. *Int. J. Dev. Neurosci.* 30 147–158. 10.1016/j.ijdevneu.2011.11.005 22155002

[B3] Allendoerfer K. L. Shatz C. J. (1994). The subplate, a transient neocortical structure: its role in the development of connections between thalamus and cortex. *Annu. Rev. Neurosci.* 17 185–218. 10.1146/annurev.ne.17.030194.001153 8210173

[B4] Altman J. Bayer S. (1975). “Postnatal development of the hippocampal dentate gyrus under normal and experimental conditions,” in *The Hippocampus*, Vol. 1 eds Isaacson R. L. Pribram K. H. (New York, NY: Plenum Press), 95–122. 10.1007/978-1-4684-2976-3_5

[B5] Altman J. Bayer S. A. (1990b). Mosaic organization of the hippocampal neuroepithelium and the multiple germinal sources of dentate granule cells. *J. Comp. Neurol.* 301 325–342. 10.1002/cne.903010302 2262594

[B6] Altman J. Bayer S. A. (1990a). Migration and distribution of two populations of hippocampal granule cell precursors during the perinatal and postnatal periods. *J. Comp. Neurol.* 301 365–381. 10.1002/cne.903010304 2262596

[B7] Altug-Teber O. Bonin M. Walter M. Mau-Holzmann U. A. Dufke A. Stappert H. (2007). Specific transcriptional changes in human fetuses with autosomal trisomies. *Cytogenet. Genome Res.* 119 171–184. 10.1159/000112058 18253026

[B8] An J. Chen B. Tian D. Guo Y. Yan Y. Yang H. (2021). Regulation of neurogenesis and neuronal differentiation by natural compounds. *Curr. Stem Cell Res. Ther.* 10.2174/1574888x16666210907141447 [Epub ahead of print]. 34493197

[B9] Angevine J. B. Jr. (1965). Time of neuron origin in the hippocampal region. An autoradiographic study in the mouse. *Exp. Neurol.* 2 1–70. 10.1016/0014-4886(65)90121-4 5838955

[B10] Antonarakis S. E. Skotko B. G. Rafii M. S. Strydom A. Pape S. E. Bianchi D. W. (2020). Down syndrome. *Nat. Rev. Dis. Primers* 6:9. 10.1038/s41572-019-0143-7 32029743 PMC8428796

[B11] Arron J. R. Winslow M. M. Polleri A. Chang C. P. Wu H. Gao X. (2006). NFAT dysregulation by increased dosage of DSCR1 and DYRK1A on chromosome 21. *Nature* 441 595–600. 10.1038/nature04678 16554754

[B12] Ash J. A. Velazquez R. Kelley C. M. Powers B. E. Ginsberg S. D. Mufson E. J. (2014). Maternal choline supplementation improves spatial mapping and increases basal forebrain cholinergic neuron number and size in aged Ts65Dn mice. *Neurobiol. Dis.* 70 32–42. 10.1016/j.nbd.2014.06.001 24932939 PMC4133151

[B13] Atas-Ozcan H. Brault V. Duchon A. Herault Y. (2021). *Dyrk1a* from gene function in development and physiology to dosage correction across life span in down syndrome. *Genes* 12:1833. 10.3390/genes12111833 34828439 PMC8624927

[B14] Baburamani A. A. Patkee P. A. Arichi T. Rutherford M. A. (2019). New approaches to studying early brain development in Down syndrome. *Dev. Med. Child Neurol.* 61 867–879. 10.1111/dmcn.14260 31102269 PMC6618001

[B15] Bahado-Singh R. O. Wyse L. Dorr M. A. Copel J. A. O’Connor T. Hobbins J. C. (1992). Fetuses with Down syndrome have disproportionately shortened frontal lobe dimensions on ultrasonographic examination. *Am. J. Obstet. Gynecol.* 167(4 Pt 1), 1009–1014. 10.1016/s0002-9378(12)80029-9 1415385

[B16] Bahn S. Mimmack M. Ryan M. Caldwell M. A. Jauniaux E. Starkey M. (2002). Neuronal target genes of the neuron-restrictive silencer factor in neurospheres derived from fetuses with Down’s syndrome: a gene expression study. *Lancet* 359 310–315. 10.1016/S0140-6736(02)07497-4 11830198

[B17] Ballard C. Mobley W. Hardy J. Williams G. Corbett A. (2016). Dementia in Down’s syndrome. *Lancet Neurol.* 15 622–636. 10.1016/S1474-4422(16)00063-6 27302127

[B18] Bardoni B. Capovilla M. Lalli E. (2017). Modeling Fragile X syndrome in neurogenesis: an unexpected phenotype and a novel tool for future therapies. *Neurogenesis* 4:e1270384. 10.1080/23262133.2016.1270384 28203608 PMC5293320

[B19] Barnes E. A. Kong M. Ollendorff V. Donoghue D. J. (2001). Patched1 interacts with cyclin B1 to regulate cell cycle progression. *Embo J.* 20 2214–2223. 10.1093/emboj/20.9.2214 11331587 PMC125436

[B20] Bayatti N. Moss J. A. Sun L. Ambrose P. Ward J. F. Lindsay S. (2008). A molecular neuroanatomical study of the developing human neocortex from 8 to 17 postconceptional weeks revealing the early differentiation of the subplate and subventricular zone. *Cereb. Cortex* 18 1536–1548. 10.1093/cercor/bhm184 17965125 PMC2430151

[B21] Beals C. R. Sheridan C. M. Turck C. W. Gardner P. Crabtree G. R. (1997). Nuclear export of NF-ATc enhanced by glycogen synthase kinase-3. *Science* 275 1930–1934. 10.1126/science.275.5308.1930 9072970

[B22] Becker L. Mito T. Takashima S. Onodera K. (1991). Growth and development of the brain in Down syndrome. *Prog. Clin. Biol. Res.* 373 133–152. 1838182

[B23] Becker L. E. Armstrong D. L. Chan F. (1986). Dendritic atrophy in children with Down’s syndrome. *Ann. Neurol.* 20 520–526. 10.1002/ana.410200413 2947535

[B24] Becker L. E. Armstrong D. L. Chan F. Wood M. M. (1984). Dendritic development in human occipital cortical neurons. *Brain Res.* 315 117–124. 10.1016/0165-3806(84)90083-x 6722572

[B25] Belichenko P. V. Kleschevnikov A. M. Salehi A. Epstein C. J. Mobley W. C. (2007). Synaptic and cognitive abnormalities in mouse models of Down syndrome: exploring genotype-phenotype relationships. *J. Comp. Neurol.* 504 329–345. 10.1002/cne.21433 17663443

[B26] Belichenko P. V. Masliah E. Kleschevnikov A. M. Villar A. J. Epstein C. J. Salehi A. (2004). Synaptic structural abnormalities in the Ts65Dn mouse model of Down Syndrome. *J. Comp. Neurol.* 480 281–298. 10.1002/cne.20337 15515178

[B27] Belyaev N. D. Kellett K. A. Beckett C. Makova N. Z. Revett T. J. Nalivaeva N. N. (2010). The transcriptionally active amyloid precursor protein (APP) intracellular domain is preferentially produced from the 695 isoform of APP in a {beta}-secretase-dependent pathway. *J. Biol. Chem.* 285 41443–41454. 10.1074/jbc.M110.141390 20961856 PMC3009870

[B28] Benavides-Piccione R. Ballesteros-Yanez I. de Lagran M. M. Elston G. Estivill X. Fillat C. (2004). On dendrites in Down syndrome and DS murine models: a spiny way to learn. *Prog. Neurobiol.* 74 111–126. 10.1016/j.pneurobio.2004.08.001 15518956

[B29] Bianchi P. Ciani E. Guidi S. Trazzi S. Felice D. Grossi G. (2010). Early pharmacotherapy restores neurogenesis and cognitive performance in the Ts65Dn mouse model for Down syndrome. *J. Neurosci.* 30 8769–8779. 10.1523/JNEUROSCI.0534-10.2010 20592198 PMC6632890

[B30] Bicker F. Nardi L. Maier J. Vasic V. Schmeisser M. J. (2021). Criss-crossing autism spectrum disorder and adult neurogenesis. *J. Neurochem.* 159 452–478. 10.1111/jnc.15501 34478569

[B31] Boldrini M. Underwood M. D. Hen R. Rosoklija G. B. Dwork A. J. John Mann J. (2009). Antidepressants increase neural progenitor cells in the human hippocampus. *Neuropsychopharmacology* 34 2376–2389. 10.1038/npp.2009.75 19606083 PMC2743790

[B32] Bonni A. Sun Y. Nadal-Vicens M. Bhatt A. Frank D. A. Rozovsky I. (1997). Regulation of gliogenesis in the central nervous system by the JAK-STAT signaling pathway. *Science* 278 477–483. 10.1126/science.278.5337.477 9334309

[B33] Brazel C. Y. Romanko M. J. Rothstein R. P. Levison S. W. (2003). Roles of the mammalian subventricular zone in brain development. *Prog. Neurobiol.* 69 49–69. 10.1016/s0301-0082(03)00002-9 12637172

[B34] Briggs J. A. Sun J. Shepherd J. Ovchinnikov D. A. Chung T.-L. Nayler S. P. (2013). Integration-free induced pluripotent stem cells model genetic and neural developmental features of down syndrome etiology. *Stem Cells* 31 467–478. 10.1002/stem.1297 23225669

[B35] Bull M. J. (2020). Down syndrome. *N. Engl. J. Med.* 382 2344–2352. 10.1056/NEJMra1706537 32521135

[B36] Busciglio J. Pelsman A. Wong C. Pigino G. Yuan M. Mori H. (2002). Altered metabolism of the amyloid beta precursor protein is associated with mitochondrial dysfunction in Down’s syndrome. *Neuron* 33 677–688. 10.1016/s0896-6273(02)00604-9 11879646

[B37] Bystron I. Blakemore C. Rakic P. (2008). Development of the human cerebral cortex: boulder committee revisited. *Nat. Rev. Neurosci.* 9 110–122. 10.1038/nrn2252 18209730

[B38] Canzonetta C. Mulligan C. Deutsch S. Ruf S. O’Doherty A. Lyle R. (2008). DYRK1A-dosage imbalance perturbs NRSF/REST levels, deregulating pluripotency and embryonic stem cell fate in Down syndrome. *Am. J. Hum. Genet.* 83 388–400. 10.1016/j.ajhg.2008.08.012 18771760 PMC2556438

[B39] Carballo G. B. Honorato J. R. de Lopes G. P. F. Spohr T. C. L. S. E. (2018). A highlight on Sonic hedgehog pathway. *Cell Commun. Signal.* 16:11. 10.1186/s12964-018-0220-7 29558958 PMC5861627

[B40] Cayuso J. Ulloa F. Cox B. Briscoe J. Marti E. (2006). The Sonic hedgehog pathway independently controls the patterning, proliferation and survival of neuroepithelial cells by regulating Gli activity. *Development* 133 517–528. 10.1242/dev.02228 16410413

[B41] Chakrabarti L. Best T. K. Cramer N. P. Carney R. S. Isaac J. T. Galdzicki Z. (2010). Olig1 and Olig2 triplication causes developmental brain defects in Down syndrome. *Nat. Neurosci.* 13 927–934. 10.1038/nn.2600 20639873 PMC3249618

[B42] Chakrabarti L. Galdzicki Z. Haydar T. F. (2007). Defects in embryonic neurogenesis and initial synapse formation in the forebrain of the Ts65Dn mouse model of Down syndrome. *J. Neurosci.* 27 11483–11495. 10.1523/JNEUROSCI.3406-07.2007 17959791 PMC6673208

[B43] Chambard J. C. Lefloch R. Pouysségur J. Lenormand P. (2007). ERK implication in cell cycle regulation. *Biochim. Biophys. Acta* 1773 1299–1310. 10.1016/j.bbamcr.2006.11.010 17188374

[B44] Chen C. Jiang P. Xue H. Peterson S. E. Tran H. T. McCann A. E. (2014). Role of astroglia in Down’s syndrome revealed by patient-derived human-induced pluripotent stem cells. *Nat. Commun.* 5:4430. 10.1038/ncomms5430 25034944 PMC4109022

[B45] Chen J. Y. Lin J. R. Tsai F. C. Meyer T. (2013). Dosage of Dyrk1a shifts cells within a p21-cyclin D1 signaling map to control the decision to enter the cell cycle. *Mol. Cell* 52 87–100. 10.1016/j.molcel.2013.09.009 24119401 PMC4039290

[B46] Chen X. Q. Salehi A. Pearn M. L. Overk C. Nguyen P. D. Kleschevnikov A. M. (2021). Targeting increased levels of APP in Down syndrome: posiphen-mediated reductions in APP and its products reverse endosomal phenotypes in the Ts65Dn mouse model. *Alzheimers Dement.* 17 271–292. 10.1002/alz.12185 32975365 PMC7984396

[B47] Cheon M. S. Bajo M. Kim S. H. Claudio J. O. Stewart A. K. Patterson D. (2003a). Protein levels of genes encoded on chromosome 21 in fetal Down syndrome brain: challenging the gene dosage effect hypothesis (Part II). *Amino Acids* 24 119–125. 10.1007/s00726-002-0337-1 12624743

[B48] Cheon M. S. Kim S. H. Ovod V. Kopitar Jerala N. Morgan J. I. Hatefi Y. (2003b). Protein levels of genes encoded on chromosome 21 in fetal Down syndrome brain: challenging the gene dosage effect hypothesis (Part III). *Amino Acids* 24 127–134. 10.1007/s00726-002-0340-6 12624744

[B49] Clancy B. Darlington R. B. Finlay B. L. (2001). Translating developmental time across mammalian species. *Neuroscience* 105 7–17. 10.1016/s0306-4522(01)00171-3 11483296

[B50] Colombo J. A. Reisin H. D. Jones M. Bentham C. (2005). Development of interlaminar astroglial processes in the cerebral cortex of control and Down’s syndrome human cases. *Exp. Neurol.* 193 207–217. 10.1016/j.expneurol.2004.11.024 15817279

[B51] Contestabile A. Fila T. Bartesaghi R. Ciani E. (2009). Cell cycle elongation impairs proliferation of cerebellar granule cell precursors in the Ts65Dn mouse, an animal model for Down syndrome. *Brain Pathol.* 19 224–237. 10.1111/j.1750-3639.2008.00168.x 18482164 PMC8094641

[B52] Contestabile A. Fila T. Ceccarelli C. Bonasoni P. Bonapace L. Santini D. (2007). Cell cycle alteration and decreased cell proliferation in the hippocampal dentate gyrus and in the neocortical germinal matrix of fetuses with Down syndrome and in Ts65Dn mice. *Hippocampus* 17 665–678. 10.1002/hipo.20308 17546680

[B53] Coronel R. Palmer C. Bernabeu-Zornoza A. Monteagudo M. Rosca A. Zambrano A. (2019). Physiological effects of amyloid precursor protein and its derivatives on neural stem cell biology and signaling pathways involved. *Neural Regen. Res.* 14 1661–1671. 10.4103/1673-5374.257511 31169172 PMC6585543

[B54] Corrales A. Parisotto E. B. Vidal V. Garcia-Cerro S. Lantigua S. Diego M. (2017). Pre- and post-natal melatonin administration partially regulates brain oxidative stress but does not improve cognitive or histological alterations in the Ts65Dn mouse model of Down syndrome. *Behav. Brain Res.* 334 142–154. 10.1016/j.bbr.2017.07.022 28743603

[B55] Corsi M. M. Dogliotti G. Pedroni F. Palazzi E. Magni P. Chiappelli M. (2006). Plasma nerve growth factor (NGF) and inflammatory cytokines (IL-6 and MCP-1) in young and adult subjects with Down syndrome: an interesting pathway. *Neuro Endocrinol. Lett.* 27 773–778. 17187019

[B56] Costa A. C. Scott-McKean J. J. (2013). Prospects for improving brain function in individuals with down syndrome. *CNS Drugs* 27 679–702. 10.1007/s40263-013-0089-3 23821040

[B57] Dang V. Medina B. Das D. Moghadam S. Martin K. J. Lin B. (2014). Formoterol, a long-acting beta2 adrenergic agonist, improves cognitive function and promotes dendritic complexity in a mouse model of Down syndrome. *Biol. Psychiatry* 75 179–188. 10.1016/j.biopsych.2013.05.024 23827853

[B58] Das I. Park J. M. Shin J. H. Jeon S. K. Lorenzi H. Linden D. J. (2013). Hedgehog agonist therapy corrects structural and cognitive deficits in a Down syndrome mouse model. *Sci. Transl. Med.* 5:201ra120. 10.1126/scitranslmed.3005983 24005160 PMC4006719

[B59] De la Torre R. De Sola S. Pons M. Duchon A. de Lagran M. M. Farre M. (2014). Epigallocatechin-3-gallate, a DYRK1A inhibitor, rescues cognitive deficits in Down syndrome mouse models and in humans. *Mol. Nutr. Food Res.* 58 278–288. 10.1002/mnfr.201300325 24039182

[B60] de Wert G. Dondorp W. Bianchi D. W. (2017). Fetal therapy for Down syndrome: an ethical exploration. *Prenat. Diagn.* 37 222–228. 10.1002/pd.4995 28004394 PMC10066512

[B61] Dierssen M. Ramakers G. J. (2006). Dendritic pathology in mental retardation: from molecular genetics to neurobiology. *Genes Brain Behav.* 5(Suppl. 2), 48–60. 10.1111/j.1601-183X.2006.00224.x 16681800

[B62] Dowjat W. K. Adayev T. Kuchna I. Nowicki K. Palminiello S. Hwang Y. W. (2007). Trisomy-driven overexpression of DYRK1A kinase in the brain of subjects with Down syndrome. *Neurosci. Lett.* 413 77–81. 10.1016/j.neulet.2006.11.026 17145134 PMC1890010

[B63] El Hajj N. Dittrich M. Bock J. Kraus T. F. Nanda I. Muller T. (2016). Epigenetic dysregulation in the developing Down syndrome cortex. *Epigenetics* 11 563–578. 10.1080/15592294.2016.1192736 27245352 PMC4990229

[B64] Emili M. Stagni F. Salvalai M. E. Uguagliati B. Giacomini A. Albac C. (2020). Neonatal therapy with clenbuterol and salmeterol restores spinogenesis and dendritic complexity in the dentate gyrus of the Ts65Dn model of Down syndrome. *Neurobiol. Dis.* 140:104874. 10.1016/j.nbd.2020.104874 32325119

[B65] Engidawork E. Gulesserian T. Fountoulakis M. Lubec G. (2003). Aberrant protein expression in cerebral cortex of fetus with Down syndrome. *Neuroscience* 122 145–154. 10.1016/s0306-4522(03)00605-5 14596856

[B66] Engidawork E. Gulesserian T. Seidl R. Cairns N. Lubec G. (2001). Expression of apoptosis related proteins: RAIDD, ZIP kinase, Bim/BOD, p21, Bax, Bcl-2 and NF-kappaB in brains of patients with Down syndrome. *J. Neural Transm. Suppl.* 61 181–192. 10.1007/978-3-7091-6262-0_14 11771742

[B67] Engidawork E. Lubec G. (2003). Molecular changes in fetal Down syndrome brain. *J. Neurochem.* 84 895–904. 10.1046/j.1471-4159.2003.01614.x 12603815

[B68] Eriksson P. S. Perfilieva E. Bjork-Eriksson T. Alborn A. M. Nordborg C. Peterson D. A. (1998). Neurogenesis in the adult human hippocampus. *Nat. Med.* 4 1313–1317. 9809557 10.1038/3305

[B69] Esposito G. Imitola J. Lu J. De Filippis D. Scuderi C. Ganesh V. S. (2008). Genomic and functional profiling of human Down syndrome neural progenitors implicates S100B and aquaporin 4 in cell injury. *Hum. Mol. Genet.* 17 440–457. 10.1093/hmg/ddm322 17984171

[B70] Ferrando-Miguel R. Shim K. S. Cheon M. S. Gimona M. Furuse M. Lubec G. (2003). Overexpression of Interferon α/β receptor β chain in fetal down syndrome brain. *Neuroembryol. Aging* 2 147–155. 10.1159/000079401

[B71] Fuentes J. J. Genesca L. Kingsbury T. J. Cunningham K. W. Perez-Riba M. Estivill X. (2000). DSCR1, overexpressed in Down syndrome, is an inhibitor of calcineurin-mediated signaling pathways. *Hum. Mol. Genet.* 9 1681–1690. 10.1093/hmg/9.11.1681 10861295

[B72] Fuerst P. G. Koizumi A. Masland R. H. Burgess R. W. (2008). Neurite arborization and mosaic spacing in the mouse retina require DSCAM. *Nature* 451 470–474. 10.1038/nature06514 18216855 PMC2259282

[B73] Garcia-Cerro S. Rueda N. Vidal V. Puente A. Campa V. Lantigua S. (2020). Prenatal administration of oleic acid or linolenic acid reduces neuromorphological and cognitive alterations in Ts65dn down syndrome mice. *J. Nutr.* 150 1631–1643. 10.1093/jn/nxaa074 32243527

[B74] Gardiner K. J. (2015). Pharmacological approaches to improving cognitive function in Down syndrome: current status and considerations. *Drug Des. Dev. Ther.* 9 103–125. 10.2147/DDDT.S51476 25552901 PMC4277121

[B75] Ge W. P. Miyawaki A. Gage F. H. Jan Y. N. Jan L. Y. (2012). Local generation of glia is a major astrocyte source in postnatal cortex. *Nature* 484 376–380. 10.1038/nature10959 22456708 PMC3777276

[B76] Giacomini A. Stagni F. Emili M. Uguagliati B. Rimondini R. Bartesaghi R. (2019). Timing of treatment with the flavonoid 7,8-dhf critically impacts on its effects on learning and memory in the Ts65Dn mouse. *Antioxidants* 8:163. 10.3390/antiox8060163 31174258 PMC6617346

[B77] Giacomini A. Stagni F. Trazzi S. Guidi S. Emili M. Brigham E. (2015). Inhibition of APP gamma-secretase restores Sonic Hedgehog signaling and neurogenesis in the Ts65Dn mouse model of Down syndrome. *Neurobiol. Dis.* 82 385–396. 10.1016/j.nbd.2015.08.001 26254735 PMC4768084

[B78] Godfrey M. Lee N. R. (2020). A comprehensive examination of the memory profile of youth with Down syndrome in comparison to typically developing peers. *Child Neuropsychol.* 26 721–738. 10.1080/09297049.2020.1721454 32100621

[B79] Golden J. A. Hyman B. T. (1994). Development of the superior temporal neocortex is anomalous in trisomy 21. *J. Neuropathol. Exp. Neurol.* 53 513–520. 10.1097/00005072-199409000-00011 8083693

[B80] Goshima T. Habara M. Maeda K. Hanaki S. Kato Y. Shimada M. (2019). Calcineurin regulates cyclin D1 stability through dephosphorylation at T286. *Sci. Rep.* 9:12779. 10.1038/s41598-019-48976-7 31484966 PMC6726757

[B81] Granato A. (2006). Altered organization of cortical interneurons in rats exposed to ethanol during neonatal life. *Brain Res.* 1069 23–30. 10.1016/j.brainres.2005.11.024 16386714

[B82] Granato A. Merighi A. (2022). Dendrites of neocortical pyramidal neurons: the key to understand intellectual disability. *Cell Mol. Neurobiol.* 42 147–153. 10.1007/s10571-021-01123-1 34216332 PMC8732981

[B83] Granese B. Scala I. Spatuzza C. Valentino A. Coletta M. Vacca R. A. (2013). Validation of microarray data in human lymphoblasts shows a role of the ubiquitin-proteasome system and NF-kB in the pathogenesis of Down syndrome. *BMC Med. Genom.* 6:24. 10.1186/1755-8794-6-24 23830204 PMC3717290

[B84] Guedj F. Siegel A. E. Pennings J. L. A. Alsebaa F. Massingham L. J. Tantravahi U. (2020). Apigenin as a candidate prenatal treatment for trisomy 21: effects in human amniocytes and the ts1cje mouse model. *Am. J. Hum. Genet.* 107 911–931. 10.1016/j.ajhg.2020.10.001 33098770 PMC7675036

[B85] Guidi S. Bonasoni P. Ceccarelli C. Santini D. Gualtieri F. Ciani E. (2008). Neurogenesis impairment and increased cell death reduce total neuron number in the hippocampal region of fetuses with Down syndrome. *Brain Pathol.* 18 180–197. 10.1111/j.1750-3639.2007.00113.x 18093248 PMC8095525

[B86] Guidi S. Ciani E. Bonasoni P. Santini D. Bartesaghi R. (2011). Widespread proliferation impairment and hypocellularity in the cerebellum of fetuses with down syndrome. *Brain Pathol.* 21 361–373. 10.1111/j.1750-3639.2010.00459.x 21040072 PMC8094247

[B87] Guidi S. Emili M. Giacomini A. Stagni F. Bartesaghi R. (2017). “Neuroanatomical alterations in the temporal cortex of human fetuses with Down syndrome,” in *Proceedings of the 2nd International Conference of the Trisomy 21 Research Society*, Chicago. 10.1111/bpa.12605

[B88] Guidi S. Giacomini A. Stagni F. Emili M. Uguagliati B. Bonasoni M. P. (2018). Abnormal development of the inferior temporal region in fetuses with Down syndrome. *Brain Pathol.* 28 986–998. 29509279 10.1111/bpa.12605 PMC8028380

[B89] Guidi S. Stagni F. Bianchi P. Ciani E. Giacomini A. De Franceschi M. (2014). Prenatal pharmacotherapy rescues brain development in a Down’s syndrome mouse model. *Brain* 137(Pt 2), 380–401. 10.1093/brain/awt340 24334313

[B90] Guidi S. Stagni F. Bianchi P. Ciani E. Ragazzi E. Trazzi S. (2013). Early pharmacotherapy with fluoxetine rescues dendritic pathology in the Ts65Dn mouse model of Down syndrome. *Brain Pathol.* 23 129–143. 10.1111/j.1750-3639.2012.00624.x 22817700 PMC8028975

[B91] Guihard-Costa A. M. Khung S. Delbecque K. Menez F. Delezoide A. L. (2006). Biometry of face and brain in fetuses with trisomy 21. *Pediatr. Res.* 59 33–38. 10.1203/01.pdr.0000190580.88391.9a 16326987

[B92] Guimera J. Casas C. Estivill X. Pritchard M. (1999). Human minibrain homologue (MNBH/DYRK1): characterization, alternative splicing, differential tissue expression, and overexpression in Down syndrome. *Genomics* 57 407–418. 10.1006/geno.1999.5775 10329007

[B93] Gutierrez-Castellanos N. Winkelman B. H. Tolosa-Rodriguez L. Devenney B. Reeves R. H. De Zeeuw C. I. (2013). Size does not always matter: Ts65Dn Down syndrome mice show cerebellum-dependent motor learning deficits that cannot be rescued by postnatal SAG treatment. *J. Neurosci.* 33 15408–15413. 10.1523/JNEUROSCI.2198-13.2013 24068809 PMC3858639

[B94] Hammerle B. Elizalde C. Galceran J. Becker W. Tejedor F. J. (2003). The MNB/DYRK1A protein kinase: neurobiological functions and Down syndrome implications. *J. Neural Transm. Suppl.* 67 129–137. 10.1007/978-3-7091-6721-2_11 15068245

[B95] Hammerle B. Ulin E. Guimera J. Becker W. Guillemot F. Tejedor F. J. (2011). Transient expression of Mnb/Dyrk1a couples cell cycle exit and differentiation of neuronal precursors by inducing p27KIP1 expression and suppressing NOTCH signaling. *Development* 138 2543–2554. 10.1242/dev.066167 21610031 PMC3100710

[B96] Hart S. J. Visootsak J. Tamburri P. Phuong P. Baumer N. Hernandez M. C. (2017). Pharmacological interventions to improve cognition and adaptive functioning in Down syndrome: strides to date. *Am. J. Med. Genet. A* 173 3029–3041. 10.1002/ajmg.a.38465 28884975

[B97] Hernandez-Gonzalez S. Ballestin R. Lopez-Hidalgo R. Gilabert-Juan J. Blasco-Ibanez J. M. Crespo C. (2015). Altered distribution of hippocampal interneurons in the murine Down Syndrome model Ts65Dn. *Neurochem. Res.* 40 151–164. 10.1007/s11064-014-1479-8 25399236

[B98] Hibaoui Y. Grad I. Letourneau A. Sailani M. R. Dahoun S. Santoni F. A. (2014). Modelling and rescuing neurodevelopmental defect of Down syndrome using induced pluripotent stem cells from monozygotic twins discordant for trisomy 21. *EMBO Mol. Med.* 6 259–277. 10.1002/emmm.201302848 24375627 PMC3927959

[B99] Hindley C. Philpott A. (2012). Co-ordination of cell cycle and differentiation in the developing nervous system. *Biochem. J.* 444 375–382. 10.1042/BJ20112040 22642576 PMC3365434

[B100] Hughes-McCormack L. A. McGowan R. Pell J. P. Mackay D. Henderson A. O’Leary L. (2020). Birth incidence, deaths and hospitalisations of children and young people with Down syndrome, 1990-2015: birth cohort study. *BMJ Open* 10:e033770. 10.1136/bmjopen-2019-033770 32241786 PMC7170621

[B101] Hutton S. R. Pevny L. H. (2011). SOX2 expression levels distinguish between neural progenitor populations of the developing dorsal telencephalon. *Dev. Biol.* 352 40–47. 10.1016/j.ydbio.2011.01.015 21256837

[B102] Incerti M. Toso L. Vink J. Roberson R. Nold C. Abebe D. (2012). Prevention of learning deficit in a Down syndrome model. *Obstet. Gynecol.* 117(2 Pt 1), 354–361. 10.1097/AOG.0b013e3182051ca5 21252750

[B103] Jiang M. Vanan S. Tu H. T. Zhang W. Zhang Z. W. Chia S. Y. (2020). Amyloid precursor protein intracellular domain-dependent regulation of FOXO3a inhibits adult hippocampal neurogenesis. *Neurobiol. Aging* 95 250–263. 10.1016/j.neurobiolaging.2020.07.031 32866886

[B104] Jung M. S. Park J. H. Ryu Y. S. Choi S. H. Yoon S. H. Kwen M. Y. (2011). Regulation of RCAN1 protein activity by Dyrk1A protein-mediated phosphorylation. *J. Biol. Chem.* 286 40401–40412. 10.1074/jbc.M111.253971 21965663 PMC3220559

[B105] Kaas J. H. (2019). The origin and evolution of neocortex: from early mammals to modern humans. *Prog. Brain Res.* 250 61–81. 10.1016/bs.pbr.2019.03.017 31703909

[B106] Kazim S. F. Blanchard J. Bianchi R. Iqbal K. (2017). Early neurotrophic pharmacotherapy rescues developmental delay and Alzheimer’s-like memory deficits in the Ts65Dn mouse model of Down syndrome. *Sci. Rep.* 7:45561. 10.1038/srep45561 28368015 PMC5377379

[B107] Kelley C. M. Powers B. E. Velazquez R. Ash J. A. Ginsberg S. D. Strupp B. J. (2014). Maternal choline supplementation differentially alters the basal forebrain cholinergic system of young-adult Ts65Dn and disomic mice. *J. Comp. Neurol.* 522 1390–1410. 10.1002/cne.23492 24178831 PMC3959592

[B108] Kenney A. M. Rowitch D. H. (2000). Sonic hedgehog promotes G(1) cyclin expression and sustained cell cycle progression in mammalian neuronal precursors. *Mol. Cell. Biol.* 20 9055–9067. 10.1128/MCB.20.23.9055-9067.2000 11074003 PMC86558

[B109] Kim S. Sato Y. Mohan P. S. Peterhoff C. Pensalfini A. Rigoglioso A. (2016). Evidence that the rab5 effector APPL1 mediates APP-βCTF-induced dysfunction of endosomes in Down syndrome and Alzheimer’s disease. *Mol. Psychiatry* 21 707–716. 10.1038/mp.2015.97 26194181 PMC4721948

[B110] Kim W. Y. Snider W. D. (2011). Functions of GSK-3 signaling in development of the nervous system. *Front. Mol. Neurosci.* 4:44. 10.3389/fnmol.2011.00044 22125510 PMC3221276

[B111] Koo B. K. Blaser S. Harwood-Nash D. Becker L. E. Murphy E. G. (1992). Magnetic resonance imaging evaluation of delayed myelination in Down syndrome: a case report and review of the literature. *J. Child Neurol.* 7 417–421. 10.1177/088307389200700417 1469252

[B112] Kostovic I. Judas M. (2010). The development of the subplate and thalamocortical connections in the human foetal brain. *Acta Paediatr.* 99 1119–1127. 10.1111/j.1651-2227.2010.01811.x 20367617

[B113] Kostović I. Sedmak G. Judaš M. (2019). Neural histology and neurogenesis of the human fetal and infant brain. *Neuroimage* 188 743–773. 10.1016/j.neuroimage.2018.12.043 30594683

[B114] Kurabayashi N. Nguyen M. D. Sanada K. (2015). DYRK1A overexpression enhances STAT activity and astrogliogenesis in a Down syndrome mouse model. *EMBO Rep.* 16 1548–1562. 10.15252/embr.201540374 26373433 PMC4641506

[B115] Kurabayashi N. Sanada K. (2013). Increased dosage of DYRK1A and DSCR1 delays neuronal differentiation in neocortical progenitor cells. *Genes Dev.* 27 2708–2721. 10.1101/gad.226381.113 24352425 PMC3877759

[B116] Lanjewar S. N. Sloan S. A. (2021). Growing glia: cultivating human stem cell models of gliogenesis in health and disease. *Front. Cell Dev. Biol.* 9:649538. 10.3389/fcell.2021.649538 33842475 PMC8027322

[B117] Larsen K. B. Laursen H. Graem N. Samuelsen G. B. Bogdanovic N. Pakkenberg B. (2008). Reduced cell number in the neocortical part of the human fetal brain in Down syndrome. *Ann. Anat.* 190 421–427. 10.1016/j.aanat.2008.05.007 18722098

[B118] Lee H. C. Tan K. L. Cheah P. S. Ling K. H. (2016). Potential Role of JAK-STAT signaling pathway in the neurogenic-to-gliogenic shift in down syndrome brain. *Neural Plast.* 2016:7434191. 10.1155/2016/7434191 26881131 PMC4737457

[B119] Li S. S. Qu Z. Haas M. Ngo L. Heo Y. J. Kang H. J. (2016). The HSA21 gene EURL/C21ORF91 controls neurogenesis within the cerebral cortex and is implicated in the pathogenesis of Down Syndrome. *Sci. Rep.* 6:29514. 10.1038/srep29514 27404227 PMC4941730

[B120] Lilja A. M. Röjdner J. Mustafiz T. Thomé C. M. Storelli E. Gonzalez D. (2013). Age-dependent neuroplasticity mechanisms in Alzheimer Tg2576 mice following modulation of brain amyloid-β levels. *PLoS One* 8:e58752. 10.1371/journal.pone.0058752 23554921 PMC3598857

[B121] Liu W. Zhou H. Liu L. Zhao C. Deng Y. Chen L. (2015). Disruption of neurogenesis and cortical development in transgenic mice misexpressing Olig2, a gene in the Down syndrome critical region. *Neurobiol. Dis.* 77 106–116. 10.1016/j.nbd.2015.02.021 25747816 PMC4428323

[B122] Lott I. T. Head E. (2019). Dementia in Down syndrome: unique insights for Alzheimer disease research. *Nat. Rev. Neurol.* 15 135–147. 10.1038/s41582-018-0132-6 30733618 PMC8061428

[B123] Lu D. He L. Xiang W. Ai W.-M. Cao Y. Wang X.-S. (2013). Somal and dendritic development of human CA3 pyramidal neurons f rom midgestation to middle childhood: a quantitative golgi study. *Anat. Rec.* 296 123–132. 10.1002/ar.22616 23152308

[B124] Lu H. E. Yang Y. C. Chen S. M. Su H. L. Huang P. C. Tsai M. S. (2013). Modeling neurogenesis impairment in Down syndrome with induced pluripotent stem cells from Trisomy 21 amniotic fluid cells. *Exp. Cell Res.* 319 498–505. 10.1016/j.yexcr.2012.09.017 23041301

[B125] Lu J. Esposito G. Scuderi C. Steardo L. Delli-Bovi L. C. Hecht J. L. (2011). S100B and APP promote a gliocentric shift and impaired neurogenesis in Down syndrome neural progenitors. *PLoS One* 6:e22126. 10.1371/journal.pone.0022126 21779383 PMC3133657

[B126] Lu J. Lian G. Zhou H. Esposito G. Steardo L. Delli-Bovi L. C. (2012). OLIG2 over-expression impairs proliferation of human Down syndrome neural progenitors. *Hum. Mol. Genet.* 21 2330–2340. 10.1093/hmg/dds052 22343408 PMC3335315

[B127] Lu Q. R. Sun T. Zhu Z. Ma N. Garcia M. Stiles C. D. (2002). Common developmental requirement for Olig function indicates a motor neuron/oligodendrocyte connection. *Cell* 109 75–86. 10.1016/s0092-8674(02)00678-5 11955448

[B128] Ly P. T. Wu Y. Zou H. Wang R. Zhou W. Kinoshita A. (2013). Inhibition of GSK3beta-mediated BACE1 expression reduces Alzheimer-associated phenotypes. *J. Clin. Invest.* 123 224–235. 10.1172/JCI64516 23202730 PMC3533290

[B129] Malik S. Vinukonda G. Vose L. R. Diamond D. Bhimavarapu B. B. R. Hu F. (2013). Neurogenesis continues in the third trimester of pregnancy and is suppressed by premature birth. *J. Neurosci.* 33 411–423. 10.1523/JNEUROSCI.4445-12.2013 23303921 PMC3711635

[B130] Marin-Padilla M. (1976). Pyramidal cell abnormalities in the motor cortex of a child with Down’s syndrome. A Golgi study. *J. Comp. Neurol.* 167 63–81. 10.1002/cne.901670105 131810

[B131] Martin K. R. Corlett A. Dubach D. Mustafa T. Coleman H. A. Parkington H. C. (2012). Over-expression of RCAN1 causes Down syndrome-like hippocampal deficits that alter learning and memory. *Hum. Mol. Genet.* 21 3025–3041. 10.1093/hmg/dds134 22511596

[B132] Masaki T. Shimada M. (2022). Decoding the phosphatase code: regulation of cell proliferation by calcineurin. *Int. J. Mol. Sci.* 23:1122. 10.3390/ijms23031122 35163061 PMC8835043

[B133] Mazur-Kolecka B. Golabek A. Kida E. Rabe A. Hwang Y. W. Adayev T. (2012). Effect of DYRK1A activity inhibition on development of neuronal progenitors isolated from Ts65Dn mice. *J. Neurosci. Res.* 90 999–1010. 10.1002/jnr.23007 22252917

[B134] Meller K. Breipohl W. Glees P. (1969). Ontogeny of the mouse motor cortex. The polymorph layer or layer VI. A Golgi and electronmicroscopical study. *Z. Zellforsch. Mikrosk Anat.* 99 443–458. 10.1007/BF00337614 4901217

[B135] Mersy E. Smits L. J. van Winden L. A. de Die-Smulders C. E. South-East Netherlands N. C. Paulussen A. D. (2013). Noninvasive detection of fetal trisomy 21: systematic review and report of quality and outcomes of diagnostic accuracy studies performed between 1997 and 2012. *Hum. Reprod. Update* 19 318–329. 10.1093/humupd/dmt001 23396607

[B136] Miranda R. C. (2012). MicroRNAs and fetal brain development: implications for ethanol teratology during the second trimester period of neurogenesis. *Front. Genet.* 3:77. 10.3389/fgene.2012.00077 22623924 PMC3353139

[B137] Montesinos M. L. (2017). Local translation of the Down syndrome cell adhesion molecule (DSCAM) mRNA in the vertebrate central nervous system. *J. Neurogenet.* 31 223–230. 10.1080/01677063.2017.1391250 29078722

[B138] Moon J. Chen M. Gandhy S. U. Strawderman M. Levitsky D. A. Maclean K. N. (2010). Perinatal choline supplementation improves cognitive functioning and emotion regulation in the Ts65Dn mouse model of Down syndrome. *Behav. Neurosci.* 124 346–361. 10.1037/a0019590 20528079 PMC2955960

[B139] Moreno-Jiménez E. P. Terreros-Roncal J. Flor-García M. Rábano A. Llorens-Martín M. (2021). Evidences for adult hippocampal neurogenesis in humans. *J. Neurosci.* 41 2541–2553. 10.1523/jneurosci.0675-20.2020 33762406 PMC8018741

[B140] Mrzljak L. Uylings H. B. Kostovic I. Van Eden C. G. (1988). Prenatal development of neurons in the human prefrontal cortex: I. A qualitative Golgi study. *J. Comp. Neurol.* 271 355–386. 10.1002/cne.902710306 2454966

[B141] Mrzljak L. Uylings H. B. Kostovic I. van Eden C. G. (1992). Prenatal development of neurons in the human prefrontal cortex. II. A quantitative Golgi study. *J. Comp. Neurol.* 316 485–496. 10.1002/cne.903160408 1577996

[B142] Murray A. Letourneau A. Canzonetta C. Stathaki E. Gimelli S. Sloan-Bena F. (2015). Brief report: isogenic induced pluripotent stem cell lines from an adult with mosaic down syndrome model accelerated neuronal ageing and neurodegeneration. *Stem Cells* 33 2077–2084. 10.1002/stem.1968 25694335 PMC4737213

[B143] Najas S. Arranz J. Lochhead P. A. Ashford A. L. Oxley D. Delabar J. M. (2015). DYRK1A-mediated Cyclin D1 degradation in neural stem cells contributes to the neurogenic cortical defects in Down syndrome. *EBioMedicine* 2 120–134. 10.1016/j.ebiom.2015.01.010 26137553 PMC4484814

[B144] Nakano-Kobayashi A. Awaya T. Kii I. Sumida Y. Okuno Y. Yoshida S. (2017). Prenatal neurogenesis induction therapy normalizes brain structure and function in Down syndrome mice. *Proc. Natl. Acad. Sci. U. S. A.* 114 10268–10273. 10.1073/pnas.1704143114 28874550 PMC5617268

[B145] Nalivaeva N. N. Turner A. J. (2013). The amyloid precursor protein: a biochemical enigma in brain development, function and disease. *FEBS Lett.* 587 2046–2054. 10.1016/j.febslet.2013.05.010 23684647

[B146] Nguyen T. L. Fruit C. Hérault Y. Meijer L. Besson T. (2017). Dual-specificity tyrosine phosphorylation-regulated kinase 1A (DYRK1A) inhibitors: a survey of recent patent literature. *Expert Opin. Ther. Pat.* 27 1183–1199. 10.1080/13543776.2017.1360285 28766366

[B147] Nho R. S. Hergert P. (2014). FoxO3a and disease progression. *World J. Biol. Chem.* 5 346–354. 10.4331/wjbc.v5.i3.346 25225602 PMC4160528

[B148] Olmos-Serrano J. L. Kang H. J. Tyler W. A. Silbereis J. C. Cheng F. Zhu Y. (2016). Down syndrome developmental brain transcriptome reveals defective oligodendrocyte differentiation and myelination. *Neuron* 89 1208–1222. 10.1016/j.neuron.2016.01.042 26924435 PMC4795969

[B149] Pan Y. Bai C. B. Joyner A. L. Wang B. (2006). Sonic hedgehog signaling regulates Gli2 transcriptional activity by suppressing its processing and degradation. *Mol. Cell Biol.* 26 3365–3377. 10.1128/mcb.26.9.3365-3377.2006 16611981 PMC1447407

[B150] Park J. Oh Y. Yoo L. Jung M. S. Song W. J. Lee S. H. (2010). Dyrk1A phosphorylates p53 and inhibits proliferation of embryonic neuronal cells. *J. Biol. Chem.* 285 31895–31906. 10.1074/jbc.M110.147520 20696760 PMC2951261

[B151] Patkee P. A. Baburamani A. A. Kyriakopoulou V. Davidson A. Avini E. Dimitrova R. (2020). Early alterations in cortical and cerebellar regional brain growth in Down Syndrome: an in vivo fetal and neonatal MRI assessment. *Neuroimage Clin.* 25:102139. 10.1016/j.nicl.2019.102139 31887718 PMC6938981

[B152] Perez-Cremades D. Hernandez S. Blasco-Ibanez J. M. Crespo C. Nacher J. Varea E. (2010). Alteration of inhibitory circuits in the somatosensory cortex of Ts65Dn mice, a model for Down’s syndrome. *J. Neural Transm.* 117 445–455. 10.1007/s00702-010-0376-9 20157742

[B153] Perez-Nunez R. Barraza N. Gonzalez-Jamett A. Cardenas A. M. Barnier J. V. Caviedes P. (2016). Overexpressed Down Syndrome Cell Adhesion Molecule (DSCAM) deregulates P21-Activated Kinase (PAK) activity in an in vitro neuronal model of down syndrome: consequences on cell process formation and extension. *Neurotox. Res.* 30 76–87. 10.1007/s12640-016-9613-9 26966010

[B154] Ponroy Bally B. Murai K. K. (2021). Astrocytes in Down syndrome across the lifespan. *Front. Cell Neurosci.* 15:702685. 10.3389/fncel.2021.702685 34483840 PMC8416355

[B155] Powers B. E. Velazquez R. Strawderman M. S. Ginsberg S. D. Mufson E. J. Strupp B. J. (2021). Maternal choline supplementation as a potential therapy for down syndrome: assessment of effects throughout the lifespan. *Front. Aging Neurosci.* 13:723046. 10.3389/fnagi.2021.723046 34690739 PMC8527982

[B156] Prinz M. Prinz B. Schulz E. (1997). The growth of non-pyramidal neurons in the primary motor cortex of man: a Golgi study. *Histol. Histopathol.* 12 895–900. 9302548

[B157] Pritchard M. Martin K. (2013). “RCAN1 and its potential contribution to the Down syndrome phenotype,” in *Down Syndrome”*, ed. Dey S. K. (London: IntechOpen), 173–205. 10.5772/52977

[B158] Purpura D. P. (1975). Normal and aberrant neuronal development in the cerebral cortex of human fetus and young infant. *UCLA Forum Med. Sci.* 18 141–169. 10.1016/b978-0-12-139050-1.50014-8 128168

[B159] Quach T. T. Stratton H. J. Khanna R. Kolattukudy P. E. Honnorat J. Meyer K. (2021). Intellectual disability: dendritic anomalies and emerging genetic perspectives. *Acta Neuropathol.* 141 139–158. 10.1007/s00401-020-02244-5 33226471 PMC7855540

[B160] Rakic P. (2003). Developmental and evolutionary adaptations of cortical radial glia. *Cereb. Cortex* 13 541–549. 10.1093/cercor/13.6.541 12764027

[B161] Rakic P. (2004). Neuroscience. Genetic control of cortical convolutions. *Science* 303 1983–1984. 10.1126/science.1096414 15044793

[B162] Rakic P. (2009). Evolution of the neocortex: a perspective from developmental biology. *Nat. Rev. Neurosci.* 10 724–735. 10.1038/nrn2719 19763105 PMC2913577

[B163] Reiche L. Göttle P. Lane L. Duek P. Park M. Azim K. (2021). C21orf91 regulates oligodendroglial precursor cell fate-a switch in the glial lineage? *Front. Cell Neurosci.* 15:653075. 10.3389/fncel.2021.653075 33796011 PMC8008080

[B164] Rice D. Barone S. (2010). Critical periods of vulnerabiliy for the developing nervpus system: evidence from humans and animal models. *Environ. Health Perspect.* 108(Suppl. 3), 511–533. 10.1289/ehp.00108s3511 10852851 PMC1637807

[B165] Roper R. J. Baxter L. L. Saran N. G. Klinedinst D. K. Beachy P. A. Reeves R. H. (2006). Defective cerebellar response to mitogenic Hedgehog signaling in Down [corrected] syndrome mice. *Proc. Natl. Acad. Sci. U. S. A.* 103 1452–1456. 10.1073/pnas.0510750103 16432181 PMC1360600

[B166] Rowitch D. H. Kriegstein A. R. (2010). Developmental genetics of vertebrate glial–cell specification. *Nature* 468 214–222. 10.1038/nature09611 21068830

[B167] Rueda N. Florez J. Dierssen M. Martinez-Cue C. (2020a). Translational validity and implications of pharmacotherapies in preclinical models of Down syndrome. *Prog. Brain Res.* 251 245–268. 10.1016/bs.pbr.2019.10.001 32057309

[B168] Rueda N. Vidal V. Garcia-Cerro S. Puente A. Campa V. Lantigua S. (2020b). Prenatal, but not postnatal, curcumin administration rescues neuromorphological and cognitive alterations in Ts65Dn Down syndrome mice. *J. Nutr.* 150 2478–2489. 10.1093/jn/nxaa207 32729926

[B169] Rueda N. Florez J. Martinez-Cue C. (2008). Effects of chronic administration of SGS-111 during adulthood and during the pre- and post-natal periods on the cognitive deficits of Ts65Dn mice, a model of Down syndrome. *Behav. Brain Res.* 188 355–367. 10.1016/j.bbr.2007.11.020 18178265

[B170] Russo C. Salis S. Dolcini V. Venezia V. Song X. H. Teller J. K. (2001). Amino-terminal modification and tyrosine phosphorylation of [corrected] carboxy-terminal fragments of the amyloid precursor protein in Alzheimer’s disease and Down’s syndrome brain. *Neurobiol. Dis.* 8 173–180. 10.1006/nbdi.2000.0357 11162251

[B171] Sachse S. M. Lievens S. Ribeiro L. F. Dascenco D. Masschaele D. Horré K. (2019). Nuclear import of the DSCAM-cytoplasmic domain drives signaling capable of inhibiting synapse formation. *Embo J.* 38:e99669. 10.15252/embj.201899669 30745319 PMC6418460

[B172] Saito Y. Oka A. Mizuguchi M. Motonaga K. Mori Y. Becker L. E. (2000). The developmental and aging changes of Down’s syndrome cell adhesion molecule expression in normal and Down’s syndrome brains. *Acta Neuropathol.* 100 654–664. 10.1007/s004010000230 11078217

[B173] Samarasinghe R. A. Miranda O. A. Buth J. E. Mitchell S. Ferando I. Watanabe M. (2021). Identification of neural oscillations and epileptiform changes in human brain organoids. *Nat. Neurosci.* 24 1488–1500. 10.1038/s41593-021-00906-5 34426698 PMC9070733

[B174] Sauvageot C. M. Stiles C. D. (2002). Molecular mechanisms controlling cortical gliogenesis. *Curr. Opin. Neurobiol.* 12 244–249. 10.1016/s0959-4388(02)00322-7 12049929

[B175] Schmidt-Sidor B. Wisniewski K. E. Shepard T. H. Sersen E. A. (1990). Brain growth in Down syndrome subjects 15 to 22 weeks of gestational age and birth to 60 months. *Clin. Neuropathol.* 9 181–190. 2146054

[B176] Seress L. Abraham H. Tornoczky T. Kosztolanyi G. (2001). Cell formation in the human hippocampal formation from mid-gestation to the late postnatal period. *Neuroscience* 105 831–843. 10.1016/s0306-4522(01)00156-7 11530221

[B177] Serrano-Perez M. C. Fernandez M. Neria F. Berjon-Otero M. Doncel-Perez E. Cano E. (2015). NFAT transcription factors regulate survival, proliferation, migration, and differentiation of neural precursor cells. *Glia* 63 987–1004. 10.1002/glia.22797 25731131

[B178] Setoguchi T. Kondo T. (2004). Nuclear export of OLIG2 in neural stem cells is essential for ciliary neurotrophic factor-induced astrocyte differentiation. *J. Cell Biol.* 166 963–968. 10.1083/jcb.200404104 15452140 PMC2172021

[B179] Shichiri M. Yoshida Y. Ishida N. Hagihara Y. Iwahashi H. Tamai H. (2011). Alpha-Tocopherol suppresses lipid peroxidation and behavioral and cognitive impairments in the Ts65Dn mouse model of Down syndrome. *Free Radic. Biol. Med.* 50 1801–1811. 10.1016/j.freeradbiomed.2011.03.023 21447382

[B180] Shu R. Wong W. Ma Q. H. Yang Z. Z. Zhu H. Liu F. J. (2015). APP intracellular domain acts as a transcriptional regulator of miR-663 suppressing neuronal differentiation. *Cell Death Dis.* 6:e1651. 10.1038/cddis.2015.10 25695604 PMC4669786

[B181] Sillitoe R. V. Joyner A. L. (2007). Morphology, molecular codes, and circuitry produce the three-dimensional complexity of the cerebellum. *Annu. Rev. Cell Dev. Biol.* 23 549–577. 10.1146/annurev.cellbio.23.090506.123237 17506688

[B182] Sobol M. Klar J. Laan L. Shahsavani M. Schuster J. Annerén G. (2019). Transcriptome and proteome profiling of neural induced pluripotent stem cells from individuals with down syndrome disclose dynamic dysregulations of key pathways and cellular functions. *Mol. Neurobiol.* 56 7113–7127. 10.1007/s12035-019-1585-3 30989628 PMC6728280

[B183] Soppa U. Schumacher J. Florencio Ortiz V. Pasqualon T. Tejedor F. J. Becker W. (2014). The Down syndrome-related protein kinase DYRK1A phosphorylates p27(Kip1) and Cyclin D1 and induces cell cycle exit and neuronal differentiation. *Cell Cycle* 13 2084–2100. 10.4161/cc.29104 24806449 PMC4111700

[B184] Souchet B. Duchon A. Gu Y. Dairou J. Chevalier C. Daubigney F. (2019). Prenatal treatment with EGCG enriched green tea extract rescues GAD67 related developmental and cognitive defects in Down syndrome mouse models. *Sci. Rep.* 9:3914. 10.1038/s41598-019-40328-9 30850713 PMC6408590

[B185] Spalding K. L. Bergmann O. Alkass K. Bernard S. Salehpour M. Huttner H. B. (2013). Dynamics of hippocampal neurogenesis in adult humans. *Cell* 153 1219–1227. 10.1016/j.cell.2013.05.002 23746839 PMC4394608

[B186] Stagni F. Giacomini A. Emili M. Guidi S. Bartesaghi R. (2018). Neurogenesis impairment: An early developmental defect in Down syndrome. *Free Radic. Biol. Med.* 114 15–32. 10.1016/j.freeradbiomed.2017.07.026 28756311

[B187] Stagni F. Giacomini A. Emili M. Trazzi S. Guidi S. Sassi M. (2016). Short- and long-term effects of neonatal pharmacotherapy with epigallocatechin-3-gallate on hippocampal development in the Ts65Dn mouse model of Down syndrome. *Neuroscience* 333 277–301. 10.1016/j.neuroscience.2016.07.031 27457036

[B188] Stagni F. Giacomini A. Emili M. Uguagliati B. Bonasoni M. P. Bartesaghi R. (2019a). Subicular hypotrophy in fetuses with Down syndrome and in the Ts65Dn model of Down syndrome. *Brain Pathol.* 29 366–379. 10.1111/bpa.12663 30325080 PMC8028278

[B189] Stagni F. Salvalai M. E. Giacomini A. Emili M. Uguagliati B. Xia E. (2019b). Neonatal treatment with cyclosporine A restores neurogenesis and spinogenesis in the Ts65Dn model of Down syndrome. *Neurobiol. Dis.* 129 44–55. 10.1016/j.nbd.2019.05.005 31085229

[B190] Stagni F. Giacomini A. Emili M. Uguagliati B. Bonasoni M. P. Bartesaghi R. (2020). Neuroanatomical alterations in higher-order thalamic nuclei of fetuses with Down syndrome. *Clin. Neurol. Neurosurg.* 194:105870. 10.1016/j.clineuro.2020.105870 32480293

[B191] Stagni F. Giacomini A. Guidi S. Ciani E. Bartesaghi R. (2015a). Timing of therapies for Down syndrome: the sooner, the better. *Front. Behav. Neurosci.* 9:265. 10.3389/fnbeh.2015.00265 26500515 PMC4594009

[B192] Stagni F. Giacomini A. Guidi S. Ciani E. Ragazzi E. Filonzi M. (2015b). Long-term effects of neonatal treatment with fluoxetine on cognitive performance in Ts65Dn mice. *Neurobiol. Dis.* 74 204–218. 10.1016/j.nbd.2014.12.005 25497735

[B193] Stagni F. Giacomini A. Guidi S. Emili M. Uguagliati B. Salvalai M. E. (2017a). A flavonoid agonist of the TrkB receptor for BDNF improves hippocampal neurogenesis and hippocampus-dependent memory in the Ts65Dn mouse model of DS. *Exp. Neurol.* 298(Pt A), 79–96. 10.1016/j.expneurol.2017.08.018 28882412

[B194] Stagni F. Raspanti A. Giacomini A. Guidi S. Emili M. Ciani E. (2017b). Long-term effect of neonatal inhibition of APP gamma-secretase on hippocampal development in the Ts65Dn mouse model of Down syndrome. *Neurobiol. Dis.* 103 11–23. 10.1016/j.nbd.2017.03.012 28359846 PMC5439029

[B195] Stagni F. Magistretti J. Guidi S. Ciani E. Mangano C. Calza L. (2013). Pharmacotherapy with fluoxetine restores functional connectivity from the dentate gyrus to field CA3 in the Ts65Dn mouse model of Down Syndrome. *PLoS One* 8:e61689. 10.1371/journal.pone.0061689 23620781 PMC3631158

[B196] Stagni F. Uguagliati B. Emili M. Giacomini A. Bartesaghi R. Guidi S. (2021). The flavonoid 7,8-DHF fosters prenatal brain proliferation potency in a mouse model of Down syndrome. *Sci. Rep.* 11:6300. 10.1038/s41598-021-85284-5 33737521 PMC7973813

[B197] Stiles J. Jernigan T. L. (2010). The basics of brain development. *Neuropsychol. Rev.* 20 327–348. 10.1007/s11065-010-9148-4 21042938 PMC2989000

[B198] Stringer M. Abeysekera I. Dria K. J. Roper R. J. Goodlett C. R. (2015). Low dose EGCG treatment beginning in adolescence does not improve cognitive impairment in a Down syndrome mouse model. *Pharmacol. Biochem. Behav.* 138 70–79. 10.1016/j.pbb.2015.09.002 26363314

[B199] Stringer M. Abeysekera I. Thomas J. LaCombe J. Stancombe K. Stewart R. J. (2017). Epigallocatechin-3-gallate (EGCG) consumption in the Ts65Dn model of Down syndrome fails to improve behavioral deficits and is detrimental to skeletal phenotypes. *Physiol. Behav.* 177 230–241. 10.1016/j.physbeh.2017.05.003 28478033 PMC5525541

[B200] Sudarov A. Joyner A. L. (2007). Cerebellum morphogenesis: the foliation pattern is orchestrated by multi-cellular anchoring centers. *Neural Dev.* 2:26. 10.1186/1749-8104-2-26 18053187 PMC2246128

[B201] Sullivan K. D. Lewis H. C. Hill A. A. Pandey A. Jackson L. P. Cabral J. M. (2016). Trisomy 21 consistently activates the interferon response. *Elife* 5:e16220. 10.7554/eLife.16220 27472900 PMC5012864

[B202] Takahashi T. Nowakowski R. S. Caviness V. S. Jr. (1996). The leaving or Q fraction of the murine cerebral proliferative epithelium: a general model of neocortical neuronogenesis. *J. Neurosci.* 16 6183–6196. 10.1523/JNEUROSCI.16-19-06183.1996 8815900 PMC6579174

[B203] Takahashi-Yanaga F. Sasaguri T. (2008). GSK-3beta regulates cyclin D1 expression: a new target for chemotherapy. *Cell Signal.* 20 581–589. 10.1016/j.cellsig.2007.10.018 18023328

[B204] Takashima S. Becker L. E. Armstrong D. L. Chan F. (1981). Abnormal neuronal development in the visual cortex of the human fetus and infant with down’s syndrome. A quantitative and qualitative Golgi study. *Brain Res.* 225 1–21. 10.1016/0006-8993(81)90314-0 6457667

[B205] Takashima S. Iida K. Mito T. Arima M. (1994). Dendritic and histochemical development and ageing in patients with Down’s syndrome. *J. Intellect. Disabil. Res.* 38(Pt 3), 265–273. 10.1111/j.1365-2788.1994.tb00394.x 8061472

[B206] Takizawa C. G. Morgan D. O. (2000). Control of mitosis by changes in the subcellular location of cyclin-B1-Cdk1 and Cdc25C. *Curr. Opin. Cell Biol.* 12 658–665. 10.1016/s0955-0674(00)00149-6 11063929

[B207] Tambalo M. Lodato S. (2020). Brain organoids: human 3D models to investigate neuronal circuits assembly, function and dysfunction. *Brain Res.* 1746:147028. 10.1016/j.brainres.2020.147028 32717276

[B208] Tang X.-Y. Xu L. Wang J. Hong Y. Wang Y. Zhu Q. (2021). DSCAM/PAK1 pathway suppression reverses neurogenesis deficits in iPSC-derived cerebral organoids from patients with Down syndrome. *J. Clin. Invest.* 131:e135763. 10.1172/JCI135763 33945512 PMC8203468

[B209] Tanner D. C. Cherry J. D. Mayer-Pröschel M. (2011). Oligodendrocyte progenitors reversibly exit the cell cycle and give rise to astrocytes in response to interferon-γ. *J. Neurosci.* 31 6235–6246. 10.1523/jneurosci.5905-10.2011 21508246 PMC3104669

[B210] Tanzi R. E. Gusella J. F. Watkins P. C. Bruns G. A. St George-Hyslop P. Van Keuren M. L. (1987). Amyloid beta protein gene: cDNA, mRNA distribution, and genetic linkage near the Alzheimer locus. *Science* 235 880–884. 10.1126/science.2949367 2949367

[B211] Tanzi R. E. McClatchey A. I. Lamperti E. D. Villa-Komaroff L. Gusella J. F. Neve R. L. (1988). Protease inhibitor domain encoded by an amyloid protein precursor mRNA associated with Alzheimer’s disease. *Nature* 331 528–530. 10.1038/331528a0 2893290

[B212] Tarui T. Im K. Madan N. Madankumar R. Skotko B. G. Schwartz A. (2020). Quantitative MRI Analyses of regional brain growth in living fetuses with Down syndrome. *Cereb. Cortex* 30 382–390. 10.1093/cercor/bhz094 31264685 PMC7029684

[B213] Tejedor F. J. Hammerle B. (2011). MNB/DYRK1A as a multiple regulator of neuronal development. *Febs J.* 278 223–235. 10.1111/j.1742-4658.2010.07954.x 21156027

[B214] Teller J. K. Russo C. DeBusk L. M. Angelini G. Zaccheo D. Dagna-Bricarelli F. (1996). Presence of soluble amyloid beta-peptide precedes amyloid plaque formation in Down’s syndrome. *Nat. Med.* 2 93–95. 10.1038/nm0196-93 8564851

[B215] ten Donkelaar H. J. Lammens M. Wesseling P. Thijssen H. O. Renier W. O. (2003). Development and developmental disorders of the human cerebellum. *J. Neurol.* 250 1025–1036. 10.1007/s00415-003-0199-9 14504962

[B216] Toso L. Cameroni I. Roberson R. Abebe D. Bissell S. Spong C. Y. (2008). Prevention of developmental delays in a Down syndrome mouse model. *Obstet. Gynecol.* 112 1242–1251. 10.1097/AOG.0b013e31818c91dc 19037032 PMC2687469

[B217] Trazzi S. Fuchs C. De Franceschi M. Mitrugno V. M. Bartesaghi R. Ciani E. (2014). APP-dependent alteration of GSK3beta activity impairs neurogenesis in the Ts65Dn mouse model of Down syndrome. *Neurobiol. Dis.* 67 24–36. 10.1016/j.nbd.2014.03.003 24636797

[B218] Trazzi S. Fuchs C. Valli E. Perini G. Bartesaghi R. Ciani E. (2013). The amyloid precursor protein (APP) triplicated gene impairs neuronal precursor differentiation and neurite development through two different domains in the Ts65Dn mouse model for Down syndrome. *J. Biol. Chem.* 288 20817–20829. 10.1074/jbc.m113.451088 23740250 PMC3774353

[B219] Trazzi S. Mitrugno V. M. Valli E. Fuchs C. Rizzi S. Guidi S. (2011). APP-dependent up-regulation of Ptch1 underlies proliferation impairment of neural precursors in Down syndrome. *Hum. Mol. Genet.* 20 1560–1573. 10.1093/hmg/ddr033 21266456

[B220] Uguagliati B. Al-Absi A. R. Stagni F. Emili M. Giacomini A. Guidi S. (2021). Early appearance of developmental alterations in the dendritic tree of the hippocampal granule cells in the Ts65Dn model of Down syndrome. *Hippocampus* 31 435–447. 10.1002/hipo.23303 33464704

[B221] Uguagliati B. Stagni F. Emili M. Giacomini A. Russo C. Guidi S. (2022). Early appearance of dendritic alterations in neocortical pyramidal neurons of the Ts65Dn model of Down syndrome. *Dev. Neurosci.* 44 23–38. 10.1159/000520925 34852343

[B222] Urbano-Gamez J. D. Casanas J. J. Benito I. Montesinos M. L. (2021). Prenatal treatment with rapamycin restores enhanced hippocampal mGluR-LTD and mushroom spine size in a Down’s syndrome mouse model. *Mol. Brain* 14:84. 10.1186/s13041-021-00795-6 34034796 PMC8152312

[B223] Vacca R. A. Bawari S. Valenti D. Tewari D. Nabavi S. F. Shirooie S. (2019). Down syndrome: neurobiological alterations and therapeutic targets. *Neurosci. Biobehav. Rev.* 98 234–255. 10.1016/j.neubiorev.2019.01.001 30615933

[B224] Valenti D. Stagni F. Emili M. Guidi S. Bartesaghi R. Vacca R. A. (2021). Impaired brain mitochondrial bioenergetics in the Ts65Dn mouse model of down syndrome is restored by neonatal treatment with the polyphenol 7,8-dihydroxyflavone. *Antioxidants* 11:62. 10.3390/antiox11010062 35052567 PMC8773005

[B225] Velazquez R. Ash J. A. Powers B. E. Kelley C. M. Strawderman M. Luscher Z. I. (2013). Maternal choline supplementation improves spatial learning and adult hippocampal neurogenesis in the Ts65Dn mouse model of Down syndrome. *Neurobiol. Dis.* 58 92–101. 10.1016/j.nbd.2013.04.016 23643842 PMC4029409

[B226] Vidal V. Garcia-Cerro S. Rueda N. Puente A. Bartesaghi R. Martinez-Cue C. (2020). Early postnatal oleic acid administration enhances synaptic development and cognitive abilities in the Ts65Dn mouse model of Down syndrome. *Nutr. Neurosci.* 10.1080/1028415X.2020.1861897 [Epub ahead of print]. 33345728

[B227] Voronova A. Fischer A. Ryan T. Al Madhoun A. Skerjanc I. S. (2011). Ascl1/Mash1 is a novel target of Gli2 during Gli2-induced neurogenesis in P19 EC cells. *PLoS One* 6:e19174. 10.1371/journal.pone.0019174 21559470 PMC3084770

[B228] Wisniewski K. E. Schmidt-Sidor B. (1989). Postnatal delay of myelin formation in brains from Down syndrome infants and children. *Clin. Neuropathol.* 8 55–62. 2524302

[B229] Wu Y. Zhang S. Xu Q. Zou H. Zhou W. Cai F. (2016). Regulation of global gene expression and cell proliferation by APP. *Sci. Rep.* 6:22460. 10.1038/srep22460 26936520 PMC4776145

[B230] Xu R. Brawner A. T. Li S. Liu J. J. Kim H. Xue H. (2019). OLIG2 drives abnormal neurodevelopmental phenotypes in human iPSC-based organoid and chimeric mouse models of Down syndrome. *Cell Stem Cell* 24 908–926.e8. 10.1016/j.stem.2019.04.014 31130512 PMC6944064

[B231] Yabut O. Domogauer J. D’Arcangelo G. (2010). Dyrk1A overexpression inhibits proliferation and induces premature neuronal differentiation of neural progenitor cells. *J. Neurosci.* 30 4004–4014. 10.1523/JNEUROSCI.4711-09.2010 20237271 PMC3842457

[B232] Yamaguchi M. Seki T. Imayoshi I. Tamamaki N. Hayashi Y. Tatebayashi Y. (2016). Neural stem cells and neuro/gliogenesis in the central nervous system: understanding the structural and functional plasticity of the developing, mature, and diseased brain. *J. Physiol. Sci.* 66 197–206. 10.1007/s12576-015-0421-4 26578509 PMC4823343

[B233] Yin X. Jin N. Shi J. Zhang Y. Wu Y. Gong C.-X. (2017). Dyrk1A overexpression leads to increase of 3R-tau expression and cognitive deficits in Ts65Dn Down syndrome mice. *Sci. Rep.* 7:619. 10.1038/s41598-017-00682-y 28377597 PMC5428843

[B234] Yun H. J. Perez J. D. R. Sosa P. Valdés J. A. Madan N. Kitano R. (2021). Regional alterations in cortical sulcal depth in living fetuses with Down syndrome. *Cereb. Cortex* 31 757–767. 10.1093/cercor/bhaa255 32940649 PMC7786357

[B235] Zdaniuk G. Wierzba-Bobrowicz T. Szpak G. M. Stêpień T. (2011). Astroglia disturbances during development of the central nervous system in fetuses with Down’s syndrome. *Folia Neuropathol.* 49 109–114. 21845539

[B236] Zhang L. Huang Y. Chen J. Y. Ding Y. Q. Song N. N. (2015). DSCAM and DSCAML1 regulate the radial migration and callosal projection in developing cerebral cortex. *Brain Res.* 1594 61–70. 10.1016/j.brainres.2014.10.060 25451118

[B237] Zhou W. B. Miao Z. N. Zhang B. Long W. Zheng F. X. Kong J. (2019). Luteolin induces hippocampal neurogenesis in the Ts65Dn mouse model of Down syndrome. *Neural Regen. Res.* 14 613–620. 10.4103/1673-5374.248519 30632501 PMC6352604

[B238] Zigman W. B. (2013). Atypical aging in Down syndrome. *Dev. Disabil. Res. Rev.* 18 51–67. 10.1002/ddrr.1128 23949829

[B239] Zigman W. B. Lott I. T. (2007). Alzheimer’s disease in Down syndrome: neurobiology and risk. *Ment. Retard. Dev. Disabil. Res. Rev.* 13 237–246. 10.1002/mrdd.20163 17910085

